# Underlying Spatial Diversity Patterns of Freshwater Crabs in Southern China, With Recommendations for Conservation of Freshwater Biodiversity

**DOI:** 10.1002/ece3.71551

**Published:** 2025-06-12

**Authors:** Boyang Shi, Xiyang Hao, Ruxiao Wang, Kelin Chu, Da Pan, Tianyu Gu, Jun Wu, Neil Cumberlidge, Hongying Sun

**Affiliations:** ^1^ Jiangsu Key Laboratory for Biodiversity and Biotechnology, College of Life Sciences Nanjing Normal University Nanjing China; ^2^ College of Life Sciences Hainan Normal University Hainan China; ^3^ Protected Areas Research Center Nanjing Institute of Environmental Sciences, Ministry of Ecology and Environment Nanjing China; ^4^ Department of Biology Northern Michigan University Marquette Michigan USA

**Keywords:** conservation priority, diversity hotspots, freshwater keystone species, nature reserves, southern China

## Abstract

Unveiling the crucial factors influencing species diversity patterns and identifying conservation priorities are recognized as essential in confronting the escalating biodiversity crisis. The south subtropical‐tropical zone of China (STZC) is a critical biodiversity area and a major economic zone. However, the conservation of freshwater biodiversity in the STZC faces severe challenges due to a lack of knowledge about the long‐term survival of keystone species. Here, we focused on freshwater crabs because they are ideal models to address such knowledge gaps due to their critical role as benthic macroinvertebrates in maintaining the functional integrity of freshwater ecosystems. We tested ecological variables that influence the distribution of identified diversity hotspots, evaluated their extent of protection in the current nature reserves (NRs), and comprehensively identified potential conservation priority areas (CPAs). Our results indicated that freshwater crab diversity was clustered in 11 diversity hotspots, which were significantly influenced by human activities (agriculture, encroachment on freshwater habitats, and urbanization), and were also evaluated in terms of local climates and landscapes. Specifically, only 5.7% of terrestrial habitats and 9.7% of the freshwater crab species were protected by NRs. Increasing the coverage of identified CPAs to either 15/30% could effectively protect nearly six to eight times the number of freshwater crab species (up to 60/82%, respectively). We highlight the potential for increasing the priority NRs and the need to protect the distributional areas of threatened species so as to maximize the future conservation of biodiversity in the increasingly threatened freshwater ecosystems in China.

## Graphical abstract

To the best of our knowledge, this is the first comprehensive study of freshwater crab diversity patterns across the south subtropical‐tropical zone of China in terms of species richness, endemism, and influencing factors, thereby offering a sound basis for the conservation of freshwater biodiversity in China. We highlight the necessity of establishing new nature reserves based on identified potential conservation priority areas, and in priority conservation areas to protect the threatened species so as to maximize the future protection of biodiversity in the increasingly threatened freshwater ecosystems in China.

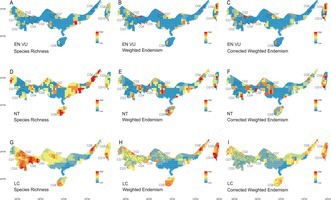

## 1 Introduction

Conservation policies and environmental impact assessments for species diversity often prioritize endangered species and habitats at high risk of extinction (Holland et al. 2012; Tsavdaridou et al. 2023; Virtanen and Moilanen 2023; Aguilar and Webb 2024). In the case of tropical and subtropical freshwater ecosystems, it is noteworthy that most conservation strategies and actions have historically focused on vertebrates, such as salamanders and amphibians (Chen et al. 2017; Liang et al. 2019; Owen et al. 2024; Velasco et al. 2024), reptiles (Francis et al. 2024; Rato et al. 2024), fishes (Abell et al. 2008; Collen et al. 2014; Tognelli et al. 2019; Koning et al. 2020; Yousefi et al. 2024), and the freshwater megafauna as a whole (Zhao et al. 2013; Braulik et al. 2024; He et al. 2024). In contrast, freshwater invertebrates have seldom been included in conservation planning globally (Collen et al. 2014; Slimani et al. 2022). Despite the fact that freshwater invertebrates and their habitats represent only a relatively small fraction of overall biodiversity (Sundar et al. 2020), they nevertheless wield significant influence over the stability of entire freshwater ecosystems (Jackson and Fuereder 2006; Geist 2011; Stendera et al. 2012; Dijkstra et al. 2014; Hill et al. 2016). Prioritizing conservation efforts focused on the study of freshwater macroinvertebrates, therefore, should be a crucial component of planning to safeguard freshwater ecosystems.

Freshwater crabs emerge as among the most ecologically significant benthic macroinvertebrates inhabiting inland waters (Rodríguez and Magalhães 2005; Dobson et al. 2007; Cumberlidge et al. 2009). These large decapod crustaceans are widely distributed from the warm temperate subtropical zone to the tropics, and with more than 1600 species recorded, they account for one‐fifth of all freshwater decapods on Earth (Cumberlidge et al. 2009; Kawai and Cumberlidge 2016; IUCN, updated by March 2024). These crabs are pivotal in upholding the functional integrity and in facilitating nutrient cycling within freshwater ecosystems (Dudgeon 2000; Strayer and Dudgeon 2010; McGeoch et al. 2011; Dijkstra et al. 2014). Moreover, the reliance of freshwater crabs on pristine freshwater conditions for their survival makes them excellent indicators of the health of inland waters (Yeo et al. 2008a; Cumberlidge et al. 2009). Nevertheless, freshwater crabs are currently confronting an unprecedented and severe crisis, primarily driven by habitat loss and pollution resulting from human activities. Alarmingly, approximately one‐sixth of extant species face an elevated risk of extinction (Yeo et al. 2008b; Cumberlidge et al. 2009; Brooks and Darwall 2011; Kawai and Cumberlidge 2016; Zeng and Yeo 2018; Shy et al. 2020; Alves et al. 2024).

The south subtropical‐tropical zone of China (STZC) has been acknowledged as a unique zoogeographical unit within Asia's biodiversity hotspots, distinguished by its exceptional biodiversity and endemism (Myers et al. 2000; Liu 2010; CEPF 2019; Hu et al. 2021). The STZC is also recognized as a key region within China's seven zoogeographical divisions, supported by quantitative national‐scale studies (Zhang 1999; Zhang and Fang 2012; He et al. 2017; Hu et al. 2021). Despite the differences between the STZC region and previous regional studies of freshwater crabs (Shih and Ng 2011; Huang, Ebach et al. 2020), there is ongoing disagreement. The STZC covers only 6% of China's total land area, yet it accounts for 16.1% of regions inhabited by freshwater crabs. Nevertheless, the STZC is still regarded as a critical hotspot for freshwater crabs, as it harbors nearly 52% of the species and 73% of the genera found in China (Dai and Cai 1998, 1999; Dai 1999; Chu et al. 2018; updated as of December 2023). This study employed the STZC by extensively referencing zoogeographical schemes, emphasizing its enhanced utility and representativeness for exploring diversity patterns of freshwater crabs compared to other regional divisions. Consequently, the freshwater crabs in the STZC serve as a valuable model for theoretical research on diversity conservation, linking spatial diversity patterns, key drivers, and biodiversity hotspots with conservation priorities.

At present, in situ conservation through the establishment of nature reserves (NRs) is widely regarded as the most rewarding strategy for biodiversity conservation (Grenyer et al. 2006; Primack 2006). In recent years, NRs in China have undergone substantial growth and development (Chi et al. 2017; Zhang et al. 2017). Since 2019, China has 15% of its land area under protective measures, with a focus predominantly centered on terrestrial flora and fauna (Xu et al. 2017; NFGA 2019). However, there is a recognized need for improvement in protecting freshwater organisms because NRs distributed in the STZC are fairly fragmented (5.7% of the STZC's land territory), with only a few targeting the protection of freshwater benthic macroinvertebrates (refer to MEE 2019). Thus, there is a need to investigate the conservation effectiveness of the NRs in the STZC and to identify priority areas for safeguarding threatened endemic freshwater crab diversity.

Here, we focus on freshwater crabs as representatives of benthic macroinvertebrates across the STZC. To explore the potential hotspots of species diversity and deepen the understanding of the conservation priorities and effectiveness of the NRs in conserving freshwater crab biodiversity, we comprehensively use species distribution models, extinction risk assessments, and spatial conservation analyses to: (1) describe the spatial distribution patterns of freshwater crab diversity, (2) investigate the key drivers influencing these distribution patterns, and (3) identify conservation gaps (CGs) and potential conservation priority areas (CPAs), especially for threatened endemic species in China.

## 2 Materials and Methods

### 2.1 Study Area and Species Occurrence Data Collection

**FIGURE 1:**
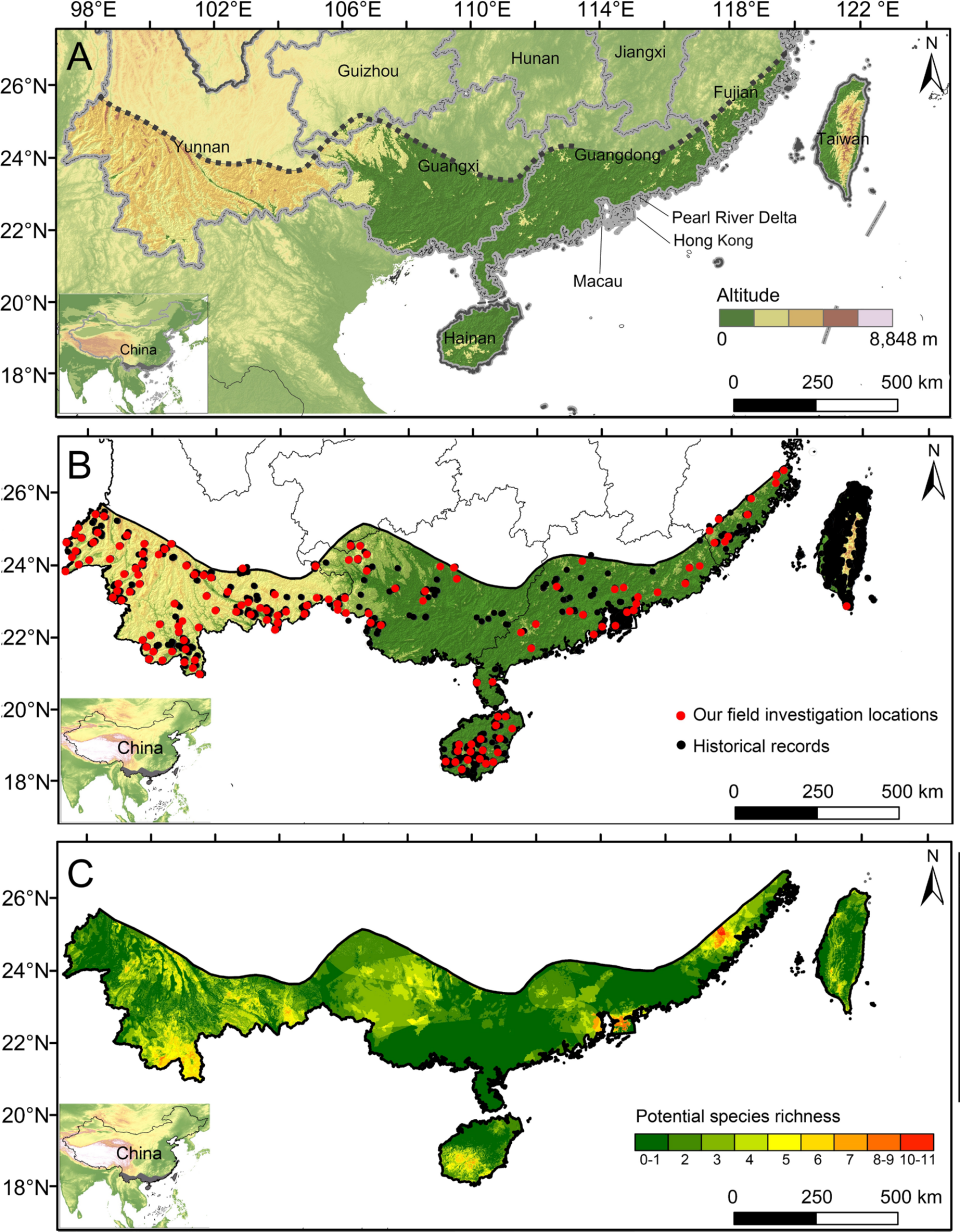
The STZC, the focal area of this study, is below the dotted line (A), with dark green representing the lowest altitudes and gray representing the highest altitudes. The distribution of freshwater crabs in the STZC (B), with red plots representing our field investigation locations and black points indicating historical distribution data. The potential species richness of freshwater crabs in the STZC (C), with dark green representing the lowest species richness and red representing the highest species richness.

The study area within the STZC encompasses nine provinces and special administrative regions, including Yunnan, Guizhou, Guangxi, Guangdong, Fujian, Taiwan, Hainan, Hong Kong, and Macau (Figure 1A). The boundary of the STZC was delineated according to the Physical Geographic Regionalization of China from the Atlas of Physical Geography of China (Liu 2010), while the national boundary of China was sourced from the National Earth System Science Data Center, National Science and Technology Infrastructure of China (http://www.geodata.cn/).

The species occurrence data were compiled by gathering research literature on freshwater crabs up to December 2023 (Table S1) and incorporating data from the Global Biodiversity Information Facility (GBIF). Furthermore, partial records are obtained from the National Parasite Resource Database (https://www.tdrc.org.cn/) and the National Animal Collection Resource Center, Institute of Zoology, Chinese Academy of Sciences, China. We further enhanced the species records with data from our long‐term field surveys conducted across the STZC from 2013 to 2024, which extend beyond previously reported locations. For historical records lacking latitude and longitude information, we converted them by establishing a 5 km buffer surrounding the described area, allowing us to include them in subsequent analyses. To ensure the accuracy of our data, we meticulously verified all spatial locations from records extracted from the literature and websites and eliminated duplicate entries and instances that significantly deviated from their original distribution sites. To mitigate potential spatial autocorrelation and address uneven sampling in our modeling analyses, we thinned the occurrence records by removing localities within a 10 km radius of each other, retaining only a single record per pixel (grid cell size: 10 × 10 km). This distance was selected based on the limited dispersal ability of freshwater crabs and has been applied in various studies (Boria et al. 2014; Kubiak et al. 2017; Bai et al. 2018; He et al. 2019). After removing duplicate georeferenced records, we retained 1131 unique records representing 195 species for further analysis (763 investigation data from our surveys and 368 historical records) (Figure 1B).

### 2.2 Species Distribution Modeling

We reconstructed the species distribution models (SDMs) to represent the geographic ranges of species, aiming to mitigate partial knowledge and inaccuracies in species distributions. Considering the semi‐aquatic life history of freshwater crabs, we incorporate bioclimatic and geographical factors to ensure a comprehensive analysis of both aquatic and terrestrial environments (Fang et al. 2013; Zeng and Yeo 2018; Daniels et al. 2024). We utilized all 19 bioclimatic variables (Bio1–19) and altitude data (Alt) at a 30 arc‐second spatial resolution (approximately 1 km) (Table S2) (downloaded from World‐Clim dataset, Stephen and Hijmans 2017). Slope (SLO) and aspect (ASP) were extracted from a 30 arc‐second gridded, quality‐controlled global Digital Elevation Model (https://data.noaa.gov).

We first identified environmental variation at each sampling location using the “raster” package (Hijmans 2016) in R v4.0 (R Core Team 2020), extracting all variables for each set of sampling coordinates. To assess redundancy among the downloaded layers, we conducted Pearson's correlation analysis using the “usdm” package (Naimi et al. 2014), and then evaluated multicollinearity by calculating the variance inflation factor (VIF) using the “vegan” package (Oksanen et al. 2020). The environmental layers with low correlation (correlation coefficient |*r*| < 0.9; Fang et al. 2013) and no significant collinearity (VIF < 5, Vicente et al. 2013; Karuno et al. 2023; Alves et al. 2024) were chosen for the following analysis. We conducted preliminary work in MaxEnt v3.4.1 to identify the most informative and influential factors, prioritizing those with a “percent contribution” greater than zero (Elith et al. 2006; Phillips et al. 2016; Phillips and Dudík 2008). Accordingly, we used a final set of 12 environmental variables to build the SDMs: ALT, SLO, ASP, Bio1, Bio2, Bio3, Bio4, Bio9, Bio10, Bio12, Bio14, and Bio15.

The SDM method was relatively insensitive to sample sizes as low as 10 (Wisz et al. 2013) and its results demonstrated explanatory power based on at least three to five occurrences (Hernandez et al. 2006; Vasconcelos et al. 2012). In this study, given the limited distribution points for freshwater crabs, we used only the maximum entropy model (MaxEnt; Phillips et al. 2006; Phillips and Dudík 2008) for SDMs, ensuring consideration of each species’ full geographic range. Additionally, by setting buffer zones around occurrence points based on known dispersal limitations (e.g., 5 km filtering in freshwater crabs), we ensured that the models did not extrapolate beyond biologically relevant areas, thereby ensuring that they truly represent SDMs rather than ENMs. For the 99 species with ≥ 4 records, we tried to establish SDMs, taking into account the time scale of historical records and adopting a relatively conservative approach. MaxEnt was used to project consensus distribution maps for these species because of its ability to handle presence‐only data and small sample sizes (*n* = 4–7) while producing highly accurate predictions (Hernandez et al. 2006; Pearson et al. 2007; Wisz et al. 2008; Rhoden et al. 2017). Furthermore, to prevent overfitting, we used the “ENMevaluate” function from the “ENMeval” package (Muscarella et al. 2014) to optimize MaxEnt models and select the best parameters (i.e., regularization multipliers and feature class) for each species. We set our modeling area to include a 5 km buffer around ≥ third‐order streams. We evaluated the models using 10‐fold cross‐validation (*k* = 10) and selected those with an Area Under the Receiver Operating Characteristic Curve (AUC) > 0.75, indicating high diagnostic accuracy.

Species with fewer than four records were addressed using a “substitution model” to mitigate potential errors in MaxEnt (*n* = 96). We projected the minimum convex polygon (MCP), constructed from all species sites, onto the sub‐basins of HydroSHEDS (Lehner and Grill 2013) (https://www.hydrosheds.org/page/hydrobasins). This dataset comprises polygonal layers delineating the boundaries of sub‐basins of different levels (1–12) in a nested manner on a global scale (Heiner et al. 2011; Tognelli et al. 2019). Subsequently, the sub‐basins representing potential distribution areas of freshwater crabs were converted into raster layers. Given that freshwater crabs on Hainan Island and Taiwan Island are constrained by the island boundaries, class 12 HydroSHEDS were applied as the replacement of the distribution areas, while class 8 was used for freshwater crab species from other areas. If the models produced by MaxEnt did not yield an AUC value exceeding 0.75, the same “substitution model” measures were taken to obtain presence‐absence binary raster files.

We conducted an average of 10 operations and exported the final results as raster files using ArcGIS v10.5 to enhance the accuracy of our operations (ESRI 2011). We employed the maximum test sensitivity and used specificity as a threshold to differentiate suitable distribution areas from unsuitable ones. The probability maps of species distribution were then converted into binary distribution maps. The adoption of a stringent threshold ensured that only highly suitable habitats were considered because commission errors (false presences) are deemed more critical than omission errors (false absences) in conservation planning (Wilson et al. 2005; McKerrow et al. 2018). The selection of this threshold method minimizes the likelihood of selecting unsuitable areas (Pearce and Ferrier 2000; Manel et al. 2001; Liu et al. 2011). Subsequently, habitats were classified as suitable or non‐suitable. Following model completion, AUC analysis was employed to evaluate simulation results (Fielding and Bell 1997), and successful modeling was defined by an AUC value not less than 0.75. Considering the distribution characteristics of freshwater crabs, binary distribution maps were clipped using a 50 km buffered MCP to exclude areas predicted to have no records. Subsequently, the resulting distributions were converted into presence‐absence binary raster layers, with a value of 0 indicating absence (predicted distributions below the adopted threshold) and 1 indicating presence (values exceeding the adopted threshold).

### 2.3 Identifying Hotspots

We overlaid all the potential distribution area maps at a 30 arc‐second spatial resolution (binary raster files) and estimated potential species richness using SDMtoolbox v2.0 (Brown et al. 2017). To ensure accuracy, we retained the most refined potential species richness map to mitigate commission errors introduced by coarse mapping methods and grain sizes (McKerrow et al. 2018). Subsequently, we conducted data classification of the summed layer to categorize values into several groups representing diverse levels of potential species richness. For the convenience of subsequent processing, we converted the classified summed raster map into polygon maps to obtain the detailed species richness data. In addition, based on the binary raster of all species, three common biodiversity indicators were estimated as follows: species richness (SR), weighted endemism (WE), and corrected weighted endemism (CWE) (Crisp et al. 2001), with the Biodiversity Measurements tool implemented in the SDMtoolbox for ArcGIS. Since the WE is correlated with the SR, we also calculated the CWE [=WE/K (the total number of species in a grid cell)] to better identify areas with low species diversity but high endemism.

In our analysis, hotspots were interpreted as areas exhibiting the highest SR and aggregation (Myers 1990; Myers et al. 2000), typically manifested by clustering conditions in spatial distributions (Chaikaew et al. 2009). Initially, Moran's I index was calculated to assess spatial autocorrelation. This inferential statistical tool determined whether or not the attribute being analyzed was randomly distributed among features in the study area (Getis and Ord 1992). A significant *p*‐value and positive z‐score reject the null hypothesis, representing random spatial processes. Subsequently, hotspots were identified based on the total number of species in each polygon. Hot Spot Analysis (Getis‐Ord Gi*) in ArcGIS was employed to calculate the GiZScore of each polygon, identifying spatial clustering of high values (hotspots) with statistical significance. Hotspots were classified based on the GiZScore values: when 1.65 < GiZScore < 1.96, 1.96 < GiZScore < 2.58, and GiZScore > 2.58; and three confidence levels (90/95/99%) of the grid's hotspots were determined, respectively (Gary et al. 2019; Ye et al. 2020).

### 2.4 Assessment of Freshwater Crabs in the STZC

The extinction risk of each of the 194 species of freshwater crabs found in the STZC was evaluated using the IUCN Red List Categories and Criteria (versions 3.1 and 14) (IUCN 2003, 2019). Species were assigned to one of eight categories to indicate their relative extinction risk: extinct (EX), extinct in the wild (EW), critically endangered (CR), endangered (EN), vulnerable (VU), near threatened (NT), least concern (LC), and data deficient (DD). Species assessed as VU, EN, or CR are described as threatened and face the highest risk of extinction in the wild. LC species are those with the lowest risk of extinction, and these are typically common and widespread. DD species are those with insufficient information to assess their extinction risk.

Most of the freshwater crab species included here were assessed primarily under Criterion B (geographic range) and Criterion D (very small or restricted population) (IUCN 2003, 2019). The extent of occurrence (EOO) and area of occupancy (AOO) of each species were used for assessment based on Criteria B1 and B2. The EOO was calculated based on SDM results for species with a disjunct distribution and limited available data and was considered the most accurate estimate (de Castro Pena et al. 2014). The AOO was determined based on the available habitat within the EOO that is actually occupied by the taxon, with each site assigned a value of 30 km^2^. Both AOO and EOO were mapped using the Geospatial Conservation Assessment Tool in ArcGIS. Meanwhile, Criterion D considers species that have a very small or restricted population. The reassessments and new assessments of the freshwater crabs reported here are all pre‐assessments intended to eventually update the most recent global assessments made in 2008 (Cumberlidge et al. 2009; IUCN 2003, 2019).

### 2.5 Correlation and Regression Analyses

To explore whether the distribution of hotspots is correlated with specific factors, we downloaded the following seven variables in addition to the 22 variables previously obtained (ALT, SLO, ASP, and Bio 1–19) (Table S2): (1) three human activity factors (crop‐agriculture, CR; human footprint index, FP; and urbanization, UR) (Sanderson et al. 2002; Wang et al. 2015); and (2) four land cover types (Forest, FR; Grassland, GR; Shrub, SH; and Others six land cover types, OT) (Finer Resolution Observation and Monitoring–Global Land Cover project, Wang et al. 2015; and the GlobeLand30 product, http://www.globallandcover.com). All variables were resampled to 1‐km resolution. Six land cover types, containing wetlands, water bodies, tundra, impervious soils, bare land with no vegetation, and snow‐covered areas, were integrated into the “others” type and excluded from the analysis due to occupying just 1% of the study area.

To avoid potential statistical issues and redundancy arising from collinearity among variables (Tabachnick et al. 2013; Huang et al. 2011), we assessed collinearity among the explanatory variables using the Pearson correlation coefficient with a common threshold of |*r*| < 0.75, employing the “usdm” package (Table S3). We also calculated the VIF for all remaining variables and confirmed that none of the retained variables had a VIF > 5 (Table S4). Consequently, the variables selected for further analysis included ALT, ASP, CR, FR, Bio1, Bio2, Bio4, Bio12, and Bio14. Pearson correlation (PC), multiple linear regression (MLR), and geographically weighted regression (GWR) were performed to examine the relationships between the species diversity index and the nine ecological variables. Unlike PC and MLR models, where a single coefficient is estimated for each explanatory variable, GWR allows for local variations in the estimation of coefficients over space. At present, the GWR model has been demonstrated to be a valuable local regression model and has increasingly been applied in various studies of species diversity (Xu et al. 2016; Tripathi et al. 2019).

We mapped the potential species diversity at 0.5° grid cells and extracted environmental variables in each 0.5° cell. The grid cells with < 50% land cover were excluded from the analysis to remove the effects of insularity, leaving 197 cells for analysis. First, we correlated the different species diversity indices using all explanatory variables. The PC analysis was performed with the R package “corrplot.” Subsequent modeling primarily focused on SR, given that this index is the most intuitive quantitative measure of diversity. In MLR, the contribution of different explanatory variables to the variation in SR was assessed using forward and backward multiple regression procedures implemented in the R package “MASS” (Venables and Ripley 2002). The Akaike information criteria (AIC) and adjusted *R* ^2^ were employed to evaluate the goodness of model fit. The model with the lowest AIC score and highest adjusted *R* ^2^ was deemed the most parsimonious (Burnham and Anderson 2002). In the GWR model, a stepwise procedure was employed to fit SR with the aforementioned eight variables. The selection of optimal bandwidth and model fitting was performed with the R package “GWmodel” (Gollini et al. 2015).

### 2.6 Conservation Priority Areas and Gaps Analysis

Diversity hotspots with confidence levels exceeding 90% were designated as priority conservation areas in order to investigate CGs and potential CPAs for freshwater crabs in the STZC. We overlaid both hotspots and NRs onto a 10 km square grid system in order to quantify the extent of coverage by NRs within hotspots. Grids identified as hotspots but lacking NR coverage were classified as CGs (Burley 1988; Scott et al. 1993; Jennings 2000).

The recently updated map of NRs in mainland China and Hainan Island was compiled from the most recent official list of NRs promulgated by the Ministry of Ecology and Environment of China, along with data from ProtectedPlanet.net (http://www.protectedplanet.net). Protected areas in Taiwan Island were sourced from the Nature Conservation Network, Forestry and Nature Conservation Agency (https://conservation.forest.gov.tw/). After excluding “marine and coastal zone” reserves, a total of 232 strict NRs and 85 protected areas in the STZC were identified. The combined area of these reserves, totaling 31,888 km^2^, accounts for approximately 5.7% of the total land area (563,710 km^2^) in the STZC.

Zonation v4.0.0 was utilized to identify spatial conservation priorities through SDMs (Moilanen et al. 2005; Lehtomäki and Moilanen 2013). This framework offers a spatial conservation prioritization methodology aimed at identifying areas crucial for maintaining habitat quality and connectivity for multiple biodiversity features simultaneously. A hierarchical priority ranking of the entire landscape was developed by iteratively removing the spatial unit (grid cell) (Moilanen et al. 2014). Selecting the core‐area Zonation (CAZ) meta‐algorithm was more appropriate than additive benefit function formulation (ABF) given that the biodiversity features in our study pertained to the potential distribution of freshwater crabs (Moilanen et al. 2014). We assigned a weighting value of 1 to species with a distribution range below the median value of all sampled species and a weighting value of 2 to those above the median value. We assigned negative weight values to pixels representing built environments in the Global Terrestrial Human Footprint map. Additionally, given that highly modified areas (e.g., built environments) tend to negatively impact freshwater habitats (Venter et al. 2016). Consequently, the sum of the weight of all biodiversity features and the negative weight value of built environments equals zero (Moilanen et al. 2014).

The current NRs were utilized as hierarchical masks to assess the proportion of freshwater species distributed within them. A warp factor of 500 and a resolution of 30 arc‐seconds (approximately 1 km) were set for all biodiversity features in Zonation. The priority map adopted the following rankings: 5.7% (the percentage of area protected by NRs in the STZC), 15% (the percentage of area protected by NRs in China), and 30% (the percentage of area protected target mentioned by COP15) (CBD 2022). The performance curve was plotted to quantify the extent to which different proportions of the landscape under priority target ranking overlap with the distribution range of species (Moilanen et al. 2014).

## 3 Results

### 3.1 Diversity Distribution Pattern and Hotspots

In the STZC, a total of 195 freshwater crab species belonging to 45 genera across 2 families were included. It is notable that despite the STZC range constituting only about 6% of the entire range in China, freshwater crabs found in the STZC encompass 73/52% of the Chinese fauna (62 genera/377 species, respectively), of which 186 species (95%) and 33 genera (73%) are endemic to China (Table S5). All species included in the distribution modeling obtained an AUC value exceeding 0.75, suggesting that the predicted results were effective.

The distribution patterns of potential diversity of the freshwater crabs in STZC reflect an uneven distribution of diversity. Regions with higher potential SR are primarily situated in central and southern Yunnan, central and western Guangxi, the Pearl River, Hong Kong, and the southwestern part of Hainan Island. Taiwan Island also demonstrates a relatively high potential SR and endemicity, but the diversity hotspots are fragmented (Figure 1C; Figure S1).

**FIGURE 2:**
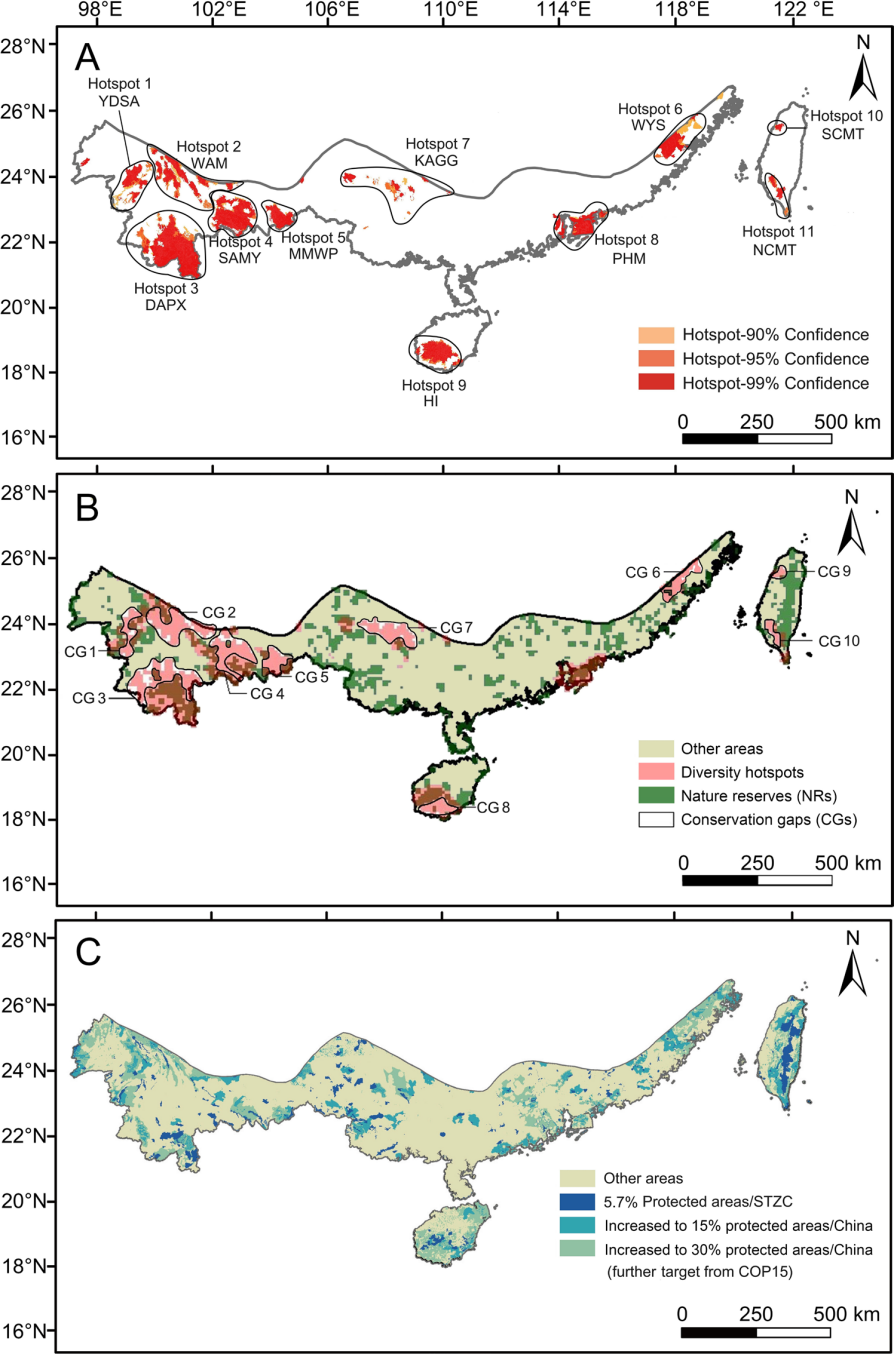
Maps showing the distribution of diversity hotspots, conservation gaps, and CPAs of freshwater crabs in the STZC. (A) 11 diversity hotspots. Hotspot 1, Yongde Daisetsuzan and the surrounding area (YDSA); Hotspot 2, Wuliang Mountains and Ailao Mountains region (WAM); Hotspot 3, Dai Autonomous Prefecture of Xishuangbanna (DAPX); Hotspot 4, southern Ailao Mountains and lower reaches of Yuanjiang River (SAMY); Hotspot 5, County‐Maguan and County‐Malipo County in Wenshan Prefecture (MMWP); Hotspot 6, Wuyishan Mountains (WYS); Hotspot 7, karst areas in Guangxi and Guizhou (KAGG); Hotspot 8, Pearl River Delta, Hong Kong, and Macau (PHM); Hotspot 9, southwestern part of Hainan Island (HI); Hotspots 10 and 11, northwestern and southern terminus of Central Mountains in Taiwan (NCMT and SCMT, respectively); (B) conservation gaps (white) between Hotspots (pink) and NRs (dark green); and (C) different types of CPAs identified in the spatial prioritization analysis covering 5.7% (dark blue), 15% (light blue), and 30% (light green) of the STZC.

The cumulative area of all 11 hotspots accounted for 13.07% of the STZC (Figure 2A), and the species found within these biodiversity hotspots are detailed in Table S6. The morphological characteristics and habitat types of freshwater crabs in various hotspot regions are detailed in Appendix S3. Five are situated in the mountains of southwest China (MSC, Hotspots 1–5). Hotspot 1 (YDSA) is located in Yongde Daisetsuzan and the surrounding area in western Yunnan, with seven genera and 11 endemic species inhabiting this area. Species in this region are primarily found at high elevations, often residing beneath rocks in mountain streams. Hotspot 2 (WAM) is distributed along the two longitudinal mountain ranges, Wuliang Mountains and Ailao Mountains, exhibiting ribbon‐like distributions. Six genera and 17 species in this area exhibit significant morphological and habitat differences.

Hotspot 3 (DAPX) encompasses most of the Dai Autonomous Prefecture of Xishuangbanna. It harbors six genera of 10 species. The region is most unique to *Tenuipotamon*, as its distribution is dominated by burrowing on the banks of small streams in the high mountains and has a smaller body size than other freshwater crabs (carapace width of the smallest mature individual = 8 mm). Hotspot 4 (SAMY), situated in the southern Ailao Mountains and lower reaches of the Yuanjiang River, includes four genera of nine stream/lake‐inhabiting species. Hotspot 5 (MMWP), located in County‐Maguan and County‐Malipo County in Wenshan Prefecture, comprises five genera of six species.

Hotspots 6–8 are situated in southeastern and south‐central China (Figure 2A). Hotspot 6 (WYS) is located in the Wuyishan Mountains, where 10 species in five genera are distributed. The first three genera are smaller in carapace size and are found under debris in streams or in burrows along banks. Hotspot 7 (KAGG) is characterized by unique karst areas in Guangxi and Guizhou, where 13 species in six genera are distributed. Several species of *Chinapotamon* have been found in the underground rivers of karst caves, while others inhabit mountain streams. Hotspot 8 (PHM) is located in the Pearl River Delta, Hong Kong, and Macau.

Hotspots 9–11 occurred on islands (Figure 2A). Hotspot 9 (HI) on the southwestern part of Hainan Island encompasses five genera and 11 species. Hotspots 10 and 11 on Taiwan Island are predominantly distributed in the northwestern and southern terminus of the Central Mountains in Taiwan (NCMT and SCMT, respectively). Hotspot 10 includes two genera with seven species. Meanwhile, Hotspot 11 comprises four genera of 18 species.

### 3.2 Conservation Status

The extinction risk of all freshwater crabs was evaluated using the IUCN Red List criteria (versions 3.1 and 14) (Table S5). Compared to the 2008 IUCN Red List of Threatened Species, the status of 82 species has changed, with eight being uplisted, six downgraded, and three reclassified as DD (Table S5). Notably, 65 species previously classified as DD have now been assigned novel statuses (2 EN, 8 VU, 30 LC, and 25 NT). Twenty species of Gecarcinucidae were assessed, of which nine species were classified as LC and eight species as NT. No species were confirmed as EN, extinct, or extinct in the wild. One hundred and seventy‐five species of Potamidae were assessed, of which 71 species were classified as LC, 51 species as NT, 11 species as EN, and 32 species as VU. No species were confirmed as extinct or extinct in the wild. Thirteen species of Chinese freshwater crabs lack comprehensive field surveys, having only one historical record available. Consequently, they are classified as DD in the assessment protocols.

### 3.3 Statistical Analysis

**FIGURE 3:**
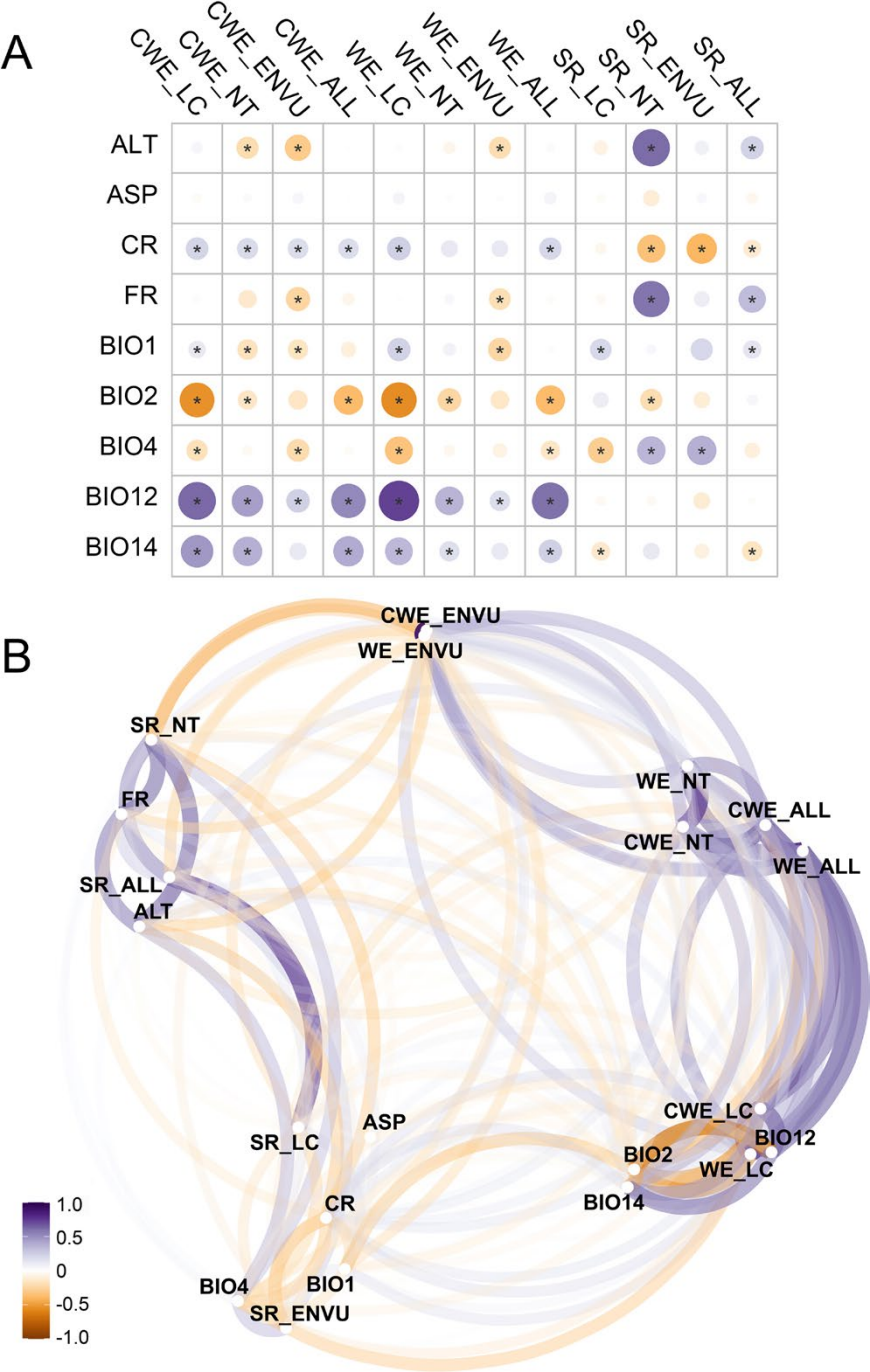
Pearson correlation coefficient (A) and correlation network (B) between the species diversity index and environmental variables. Endangered (EN), vulnerable (VU), near threatened (NT), and least concern (LC). Species richness of all species/EV VUspecies/NT species/LC species (SR_ALL/ENVU/NT/LC), weighted endemism of all species/EV VUspecies/NT species/LC species (WE_ALL/ENVU/NT/LC), corrected weighted endemism of all species/EV VUspecies/NT species/LC species (CWE_ALL/ENVU/NT/LC). Asterisks indicate values with associated *p*‐values < 0.05. The abbreviations of the explanatory variables are shown in Table S2.

The correlation analysis revealed significant correlations between the SR and specific factors (positive, +; negative, −; *p* < 0.01) (Figure 3A): temperature (annual mean temperature (+) and mean temperature of driest/coldest quarter (+)), precipitation (precipitation seasonality (+) and precipitation of driest/coldest quarter (−)), landscape (altitude (+), slope (−)), human activities (crop (−), footprint (−), urban (−)), and land cover (forest (+)). However, species endemicity exhibited a significant correlation that was opposite to SR, characterized by lower temperatures, higher rainfall, less forest coverage, greater human activity, and higher endemism (Figure 3A). The correlation network analysis highlighted that altitude and agriculture had the most pronounced impact on species diversity, whereas precipitation exhibited a more substantial effect on species endemism (Figure 3B).

The MLR model with the lowest Akaike Information Criterion corrected (AICc) value was the mixed model, incorporating landscape, land cover, temperature, and precipitation. The relationship between SR and environmental variables was determined with five independent variables based on the MLR model, which provided a significant fit for species diversity (AICc = 856.910; Multiple *R* ^2^ = 0.355, Adjusted *R* ^2^ = 0.347), that is, 48% of the variation in freshwater crab SR can be explained by these environmental variables. The potential SR of freshwater crabs in STZC was positively associated with altitude (+), forest (+), annual mean temperature (+), and (*t* = 2.234–7.048, *p* < 0.01), but negatively associated with crop (−) and precipitation of driest month (−) (*t* = −3.442 to −2.341, *p* < 0.01) (Table S7).

Comparatively, the GWR model demonstrated superior model fits (AICc = 784.749; Adjusted *R* ^2^ = 0.671; Table S8). Higher adjusted *R* ^2^, lower AICc, and Residual Sum of Squares (RSS) values indicated that the GWR model was more explanatory than the MLR model for examining the relationships between the richness patterns of freshwater crabs and environmental variables in the STZC. The GWR results highlighted crop cultivation, altitude, forest coverage, and annual mean temperature as critical drivers influencing species diversity and endemicity (Table S9).

### 3.4 Identification of CPAs and Conservation Gaps

CPAs were identified that cover wide ranges and are scattered within areas adjacent to current NRs. Upon comparison with the distribution of the previously identified hotspots, a few of the CPAs were located within the diversity hotspots, while the majority were situated outside the hotspots (Figure 2B,C). Ten CGs were identified that were widely distributed within recognized hotspots except for Hotspot 7 (Figure 2B). Seven of these CGs are located in the mainland, including MSC (CG 1–5), WYS (CG 6), and KAGG (CG 7). The remaining three were situated on islands, including HI (CG 8), SCMT, and NCMT (CG 9 and 10).

**FIGURE 4:**
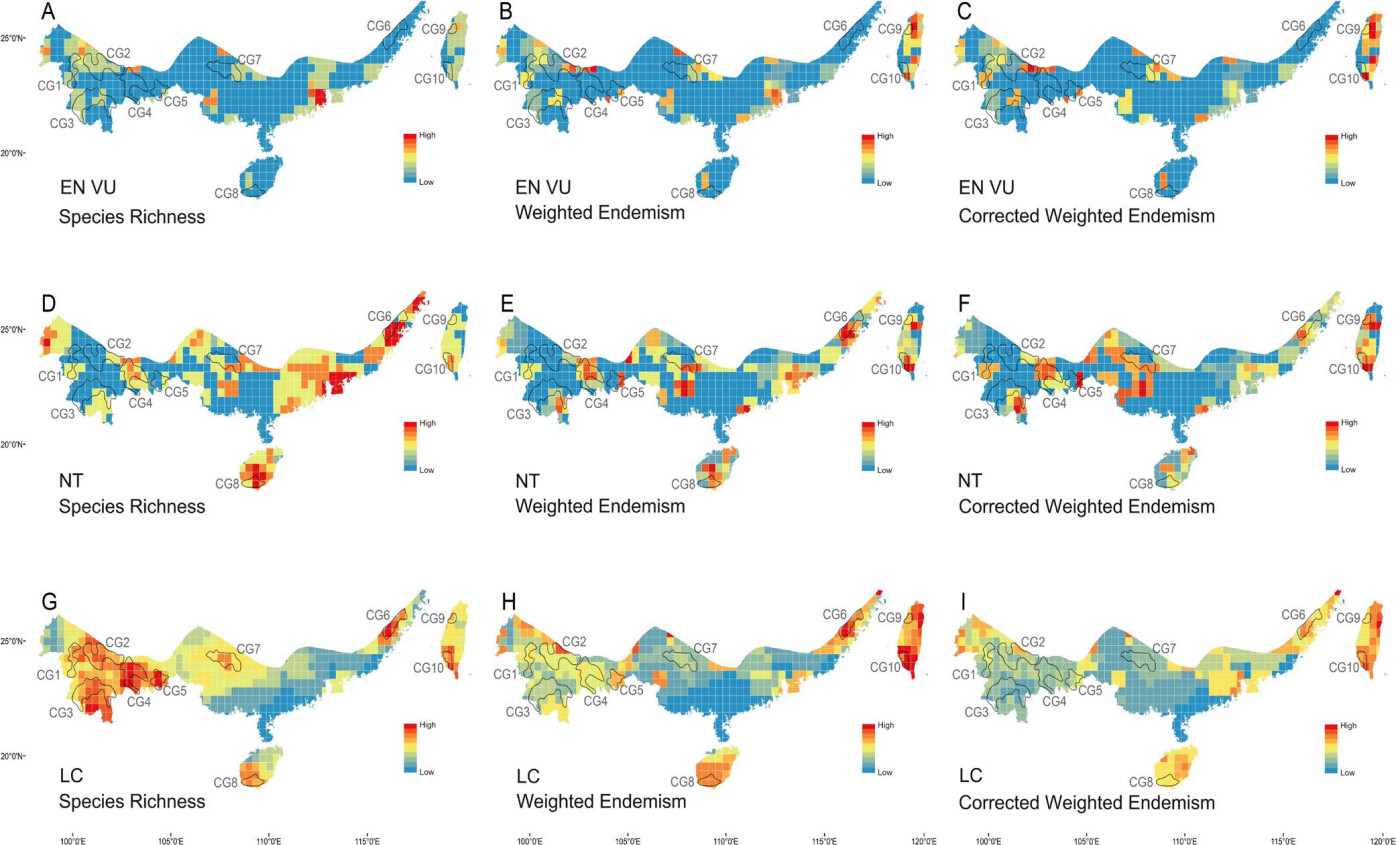
Species richness, weighted endemism, and corrected weighted endemism patterns of different assessment statuses of freshwater crabs in the STZC. Endangered and vulnerable (EN VU), near threatened (NT), and least concern (LC). Conservation gap 1–10, CG 1–10. (A–C) EN and VU species; (D–F) NT species; and (G–I) LC species.

Reassessments of species using updated data produced different assessment results and revealed that the priority conservation areas for EN and VU species were mainly concentrated at locations WAM (CG 3), SAMY (CG 4), SCMT (CG 9), and NCMT (CG 10) (Figure 4A–C). For NT species, diversity is focused on DAPX (CG 2), WYS (CG 7), KAGG (CG 6), and HI (CG 8) (Figure 4D–F). LC species were primarily concentrated in the mountains of southern Yunnan and Wuyishan, including YDSA (CG 1) and MMWP (CG 5) (Figure 4G–I).

**FIGURE 5:**
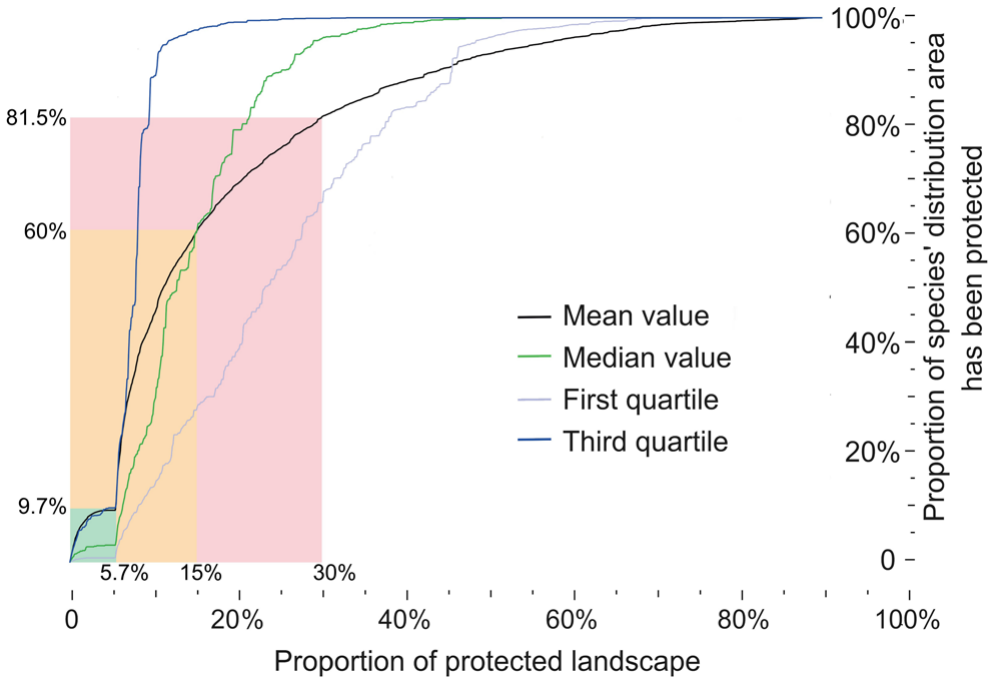
Performance curves on coverage for freshwater crab species. The curve shows the proportion of grids that are protected (x‐axis) and the corresponding average species range that is protected (y‐axis). The green box indicates the CPAs covered by the current NRs in the STZC; the orange box represents CPAs covering 15% (the percentage of the area protected by NRs in China); and the red box represents CPAs covering 30% (the percentage of the area protected target mentioned by COP15).

Currently, only about 5.7% of the surface area of STZC is protected by current NRs (Figures 2C, 5; Figure S2), which means that only 9.7% of the distributional areas of freshwater crabs are protected by the NRs. The prioritization analyses indicated that an increase of 9.3%/24.7% (about 52,400 km^2^), representing the percentage of all current NRs in China, would contribute to a significant increase in the average species range protected from 9.7% to 60%/81.5% (Figure 5; Figure S2).

## 4 Discussion

### 4.1 Determinants of Biodiversity Hotspots

Mountains play a crucial role in conserving biodiversity by preserving biota during prolonged harsh environmental conditions, and by facilitating in situ rapid diversification due to habitat discontinuity or fragmentation (Favre et al. 2015; Rahbek et al. 2019; Li et al. 2021; Zhang et al. 2022; He et al. 2023; Luo et al. 2024). The sky islands represent a unique unit in the MSC regions (Hotspots 1–5). These mountains provide high‐altitude habitats for montane species, and their deep river valleys have strong downslope windstorms (foehn winds) that increase the temperature in the valleys and contribute to their acting as geographic barriers to dispersal (Pan et al. 2012, 2022; He and Jiang 2014; Shi et al. 2024). Meanwhile, mountains can also serve as refuges for freshwater crabs, ultimately promoting phylogeographic isolation and genetic differentiation (Fang et al. 2015; Afzali et al. 2024; Mengel and Daniels 2024; Shi et al. 2021, 2024). The majority of typical mountain‐dwelling freshwater crabs in the region are concentrated in restricted island habitats at different elevations whose isolation promotes species differentiation and diversification (Figure 2A), while altitude may serve as a refuge but risks isolating populations. Freshwater crab diversity at WYS (Hotspot 6) may have been significantly affected by paleoclimatic changes rather than by elevation alone. Since the Neoproterozoic, the intensification of the East Asian monsoon has resulted in increased rainfall in eastern China (Clift et al. 2008; Li et al. 2021) that created new ecological opportunities for species radiation (Chen et al. 2018). It is hypothesized that this intensified climatic change contributes to the accumulating diversity of WYS.

Unlike mainland mountain ecosystems, the KAFF (Hotspot 7) may be influenced by the locally unique karst landscape (Figure 2A). This area features one of the most concentrated karst landform groups in China comprising cones, towers, depressions, caves, tiankengs, and other diverse forms of geographical terrain that constitute fragmented, isolated island‐like habitats, each with unique ecosystem conditions that result in species diversity (Li et al. 2013; Kong et al. 2017). Recently, numerous cavernicolous crabs with an extremely narrow distribution have been reported from the KAFF (Hotspot 7) (e.g., the genera *Chinapotamon* and *Phasmon*) (Ng 2017; Huang, Ahyong et al. 2020). Additionally, species like *Tiwaripotamon* are found in rock crevices on mountain sides (Ng and Yeo 2001; Van Tu Do and Huang 2016). Despite these recent discoveries, it is likely that the diversity of this region is still underestimated due to the large number of unexplored habitats in KAGG.

PHM (Hotspot 8) is situated in one of the most densely populated and economically developed areas in China. Species found here typically inhabit small hill streams, and many are confined to islands, such as *Cantopotamon hengqinense* and *Nanhaipotamon guangdongense* (Huang et al. 2012, 2017, 2018). *Cantopotamon hengqinense* has been assessed as “endangered” due to significant urbanization and limited forest coverage in its habitat (Huang et al. 2018). It is important, therefore, to focus attention on these threatened species that have restricted ranges that are threatened by rapid economic development.

Hotspot 9 on Hainan Island may be influenced by periodic sea level fluctuations resulting from Pleistocene glacial‐interglacial cycles. During past warmer interglacial periods when sea levels were higher than today, the high‐altitude mountains in south‐central Hainan Island served as refuges for terrestrial species (Chou et al. 2007; Hu et al. 2018). As the climate cooled and sea levels fell, some freshwater crabs on these islands expanded from high‐altitude mountainous areas to lower‐altitude mountain hills and basins, eventually reaching the exposed alluvial plains and coastal plains. Subsequently, when the sea levels rose again, these species retreated into the higher elevations of the mountains, leading to contraction–expansion cycles of their distributional ranges corresponding to glacial‐interglacial oscillations. Overall, the contraction and expansion of habitats have driven the diversification of freshwater crabs by promoting the development of distinct populations with unique adaptations.

Taiwan is renowned for its high biodiversity and endemism (Lei et al. 2003; Feng et al. 2016; He et al. 2018), and the local fauna has been significantly influenced by geological processes, particularly the collision of the Philippine Sea Plate and the Eurasian Plate around 5 million years ago (~5 Ma) (Teng 1990; Sibuet and Hsu 2004). Recent evolutionary studies indicate that geological events have driven the in situ colonization and divergence of Taiwanese freshwater crabs (Shih et al. 2007, 2011). Taiwan's diverse habitats and significant elevational variations support a wide range of species (Chou et al. 2011; Chiang et al. 2010, 2012; Ito et al. 2017). Therefore, these factors are crucial for enhancing the diversity of local freshwater crabs, which also contribute to the richness of Hotspot 10 (northern Taiwan) and Hotspot 11 (southern Taiwan). Future targeted distribution studies, combined with investigations of other aquatic invertebrates, could more effectively elucidate the factors contributing to these diversity hotspots.

### 4.2 Diversity and Endemism Responses to Human Activities and Climatic Changes

Understanding spatial diversity responses to human activities and climatic changes is fundamental for effective biodiversity conservation (Lu et al. 2020; Bernard et al. 2024; Yang et al. 2024). Importantly, human activities adjacent to large natural areas, such as near the remaining tropical rainforests in Yunnan, Hainan, and Guangxi provinces, are likely to expand in the future, resulting in increasing threats to freshwater ecosystems and their faunas. The significant linear relationship between SR and land cover links changes in land use (such as the expansion of urbanization) with a reduction in freshwater crab diversity (Figure 3). Moreover, human activities have facilitated the dispersal of numerous species well beyond their native ranges by bridging natural barriers to dispersal (Forister et al. 2021; Boggess et al. 2024). This phenomenon might also contribute to changes in the endemism of freshwater crabs if these species were to be transported from their native habitat to new areas.

The type and stability of land cover are heavily dependent on human activities such as forest harvesting and grazing that change land use, drainage patterns, and vegetation cover (Jiang et al. 2021). For example, the primary driver of biodiversity degradation since the 1990s has been the expansion of agriculture (Mantyka‐Pringle et al. 2016; Yin et al. 2020). The conversion of natural land to agriculture typically has a negative impact on freshwater ecosystems (van Soesbergen et al. 2019; Alves et al. 2024). In mountainous areas like hotspot 1 (YDSA), 2 (WAM), 3 (DAPX), and 7 (KAGG) within the STZC, human activities have had less impact, resulting in the persistence of a complex of natural diversity patterns (Figure 3). Conversely, low‐altitude areas with low freshwater crab diversity often have a large proportion of cultivated land. Since 1980, rice yields have increased by over 30% in most provinces, and corn yields have doubled or tripled in Yunnan and Chongqing provinces (Li et al. 2019). Habitat‐specialist species are more likely to be negatively impacted by anthropogenic habitat loss, such as that caused by agriculture and urbanization (Owens and Bennett 2000; Luría‐Manzano et al. 2024). In this study, the negative impacts of agriculture and urbanization further underscore the urgency of adopting sustainable practices and establishing buffer zones. In addition, in the case of widely distributed organisms, such as some species of freshwater crabs, it is important to address the harm caused by human land use as well as focus on protecting specific habitats. It is essential, therefore, to actively consider the impact of agricultural land use on key species found in protected areas and to carefully balance the relationship between food security and biodiversity.

Variations in temperature and precipitation also influence patterns of species diversity and endemism in freshwater crabs because their reliance on aquatic habitats makes them highly sensitive to environmental conditions (Jesse et al. 2011; Fang et al. 2013; Shi et al. 2021). For example, freshwater crabs become inactive and retreat into sheltered parts of their habitat during the driest and coldest parts of the year (Dai 1999). In contrast, areas with higher temperatures and lower precipitation levels tend to favor species survival and contribute to the accumulation of diversity. In addition, the climatic variables that regulate SR and endemism differ, with endemism patterns being most strongly affected by topographic heterogeneity (Schuldt and Assmann 2011; Sosa and Loera 2017; Noroozi et al. 2018), which is consistent with the results of our study.

Sharp climatic transitions are closely linked to extinction risk and impose significant physiological constraints on freshwater crabs, as their narrow climatic niches make them particularly vulnerable. Ectotherm metabolism is more dependent on climate than that of endothermic vertebrates, making variations in temperature and water availability major limiting factors for activity and embryonic development (Buckley et al. 2012; Cunningham et al. 2016; Ma et al. 2018; Falaschi et al. 2023). Freshwater crabs inhabiting regions with lower temperatures and pronounced climatic seasonality (e.g., precipitation change) tend to face a higher risk of extinction, highlighting the need to monitor areas vulnerable to climate change. In addition, the positive link with forest cover underscores the importance of preserving stable natural habitats as refuges for enduring species. Overall, these patterns suggest that external factors—both climatic and anthropogenic—can act independently or synergistically with intrinsic biological traits to drive species toward an increased risk of extinction.

### 4.3 Spatial Conservation Priorities

NRs that are specially protected and managed are the cornerstones of biodiversity conservation (Ma et al. 2019). Currently, NRs cover 30% of China's land area (Wei et al. 2021), which exceeds the 17% target set out in Aichi Target 11 for terrestrial areas (CBD 2010, 2022). However, while 34% of the current hotspots of freshwater species are found inside NRs in the 10 km grid system (Figure 2B,C), there are still many CGs where threatened species receive little or no protection. Therefore, even though Aichi Target 11 has been met, there is still a need to add more protected areas in the future. Meanwhile, the targeted conservation planning that considers CGs could provide more adequate protection for freshwater crab hotspots.

Identification of CPAs based on both biodiversity hotspots and CGs is, therefore, an important prerequisite for guiding decision‐makers in the implementation of regional ecological restoration projects (Zhang et al. 2021; Qin et al. 2022; Yin et al. 2022; Lamounier et al. 2024). Our results show that CPAs overlap well with the centers of Chinese freshwater crab SR, but that only a few of the high‐priority regions were conserved in protected areas. Only a few species of freshwater crabs are currently protected by existing NRs, whereas 43% of all 154 focal species that have distributional ranges smaller than 1300 km^2^ are found in the priority areas identified here (Figure S2). This underscores that the designated CPAs will have a greater conservation impact on species with a limited range.

Critical conservation measures include increasing the size of reserves based on CPAs in order to cover more CGs (Chi et al. 2017; Xue et al. 2021; Zhao et al. 2023). It is often impractical to fill all CGs, but CPAs that include multi‐species conservation planning based on existing protected areas can be effective in protecting species (Nori et al. 2016; Ramírez‐Albores et al. 2021). Our results suggest that expanding existing reserves based on CGs would cover approximately 10% to 25% of the total area of the STZC and would significantly benefit freshwater crab conservation. This expansion would result in a six‐fold increase in the average species‐protected range, from 9.7% to 60% and 81.5% (Figure 5). Therefore, identifying and filling obvious and aggregated CGs between CPAs and NRs is critical to conserving biodiversity in the STZC. In China, most habitats are highly fragmented and scattered, which is evident in the numerous small NRs (CBD 2010, 2022). Therefore, integrating CPAs into land‐use planning is crucial for developing effective national policies for ecological conservation, as it helps align ecological sustainability with human activities. Furthermore, given the complexity and long‐term nature of future scenarios (Liang et al. 2018), future research should consider incorporating policy mechanisms, such as ecological compensation and community‐based conservation, to optimize ecological conservation under various environmental change scenarios (Berkes 2004; Frayer et al. 2014; Sonter et al. 2020; Zhu et al. 2023).

Additionally, it is essential to consider information about different Red List categories of each taxon in the IUCN when planning conservation efforts, because species face a much higher extinction risk than currently realized (Cumberlidge et al. 2009; Kawai and Cumberlidge 2016). With over 370 freshwater crabs, China hosts the highest diversity in the world, with 95% being endemic (update to 2024). However, only 265 of these species have been assessed according to the IUCN Red List criteria, and 173 are classified as DD. While approximately 6.4% of freshwater crabs in China—including those from Macao, Hong Kong, and Taiwan—are assessed as endangered (EN), this is likely an underestimate due to reliance on outdated data (prior to 2008) and the significant proportion of species categorized as DD (over 70%) (Cumberlidge et al. 2009, 2011).

In this study, 41 (23%) of the 182 non‐DD freshwater crab species from the STZC are currently assessed as being at risk of global extinction, raising concerns about the long‐term survival of the continent's largely endemic freshwater crab fauna. It is critical to recognize that species with a small distribution range can be highly important locally and are likely to be at a greater risk of extinction (Gaston and Fuller 2009; Martínez‐Núñez et al. 2023). Nevertheless, endemic threatened freshwater crab species have limited distributions in Hotspots 1 (YDSA), 2 (WAM), 3 (DAPX), 7 (KAGG), and 11 (NCMT) (Figure 4; Table S6). These species also hold significant ecological value, emphasizing the urgent need for enhanced conservation efforts. Consequently, in relation to other conservation priorities (Figure 2C), areas supporting endangered species are strongly recommended as primary conservation targets (e.g., the five hotspots previously mentioned). Additionally, the freshwater crabs of the STZC exhibit a high degree of endemism, with many species inhabiting specialized habitats such as river rapids, lowland marshes, forested highlands, islands, water‐filled crevices in limestone mountains, and stygomorphic caves (please see Appendix S3 for further habitat details). It is also recommended to strengthen protection efforts and prioritize species with limited distribution ranges or those residing in unique habitats.

### 4.4 Implications for Further Biodiversity Conservation

The conservation of biodiversity in STZC fundamentally depends on the successful establishment and management of NRs. Although the existing NRs in STZC protect many species of freshwater crabs, our regional‐level analyses show that the current NRs in STZC do not offer protection for the majority of freshwater crabs in STZC. Nevertheless, increasing the coverage of CPAs to either 15% or 30% could effectively protect nearly eight times the number of Chinese freshwater crab species (up to 60% or 81.5%, respectively) and would represent a significant improvement in their conservation. At present, there are no well‐founded answers to how much freshwater crab habitat to protect at the regional or national levels. Given the growing threats to natural ecosystems, the most critical issue is to specify the minimum number of protected areas–N% (Tian et al. 2019). The Post‐2020 Global Biodiversity Framework produced at the 15th Conference of the Parties to the Convention on Biological Diversity (COP‐15) indicates that an increase in the protected areas in China would improve the conservation of freshwater crabs significantly.

Noteworthy, this research represents the first attempt to utilize a novel analytical approach to identify CGs and priorities for freshwater crabs. Initially, we developed stacked SDMs based on all available records of freshwater crabs, including estimating the potential distribution areas of DD species using the “substitution model” based on sub‐basins (Sayer et al. 2018). The identification here of potential diversity patterns and CPAs has provided an effective framework capable of clarifying the crucial areas in need of protection and diversity restoration. These findings can provide valuable information on the development of protection and can serve as a practical decision‐making tool and theoretical framework for future freshwater biodiversity conservation efforts. In addition, the diversity of individual regions may be underestimated due to the lack of detailed field surveys, such as those conducted in Taiwan and Guangxi. Further studies that combine a quantitative national‐scale investigation with high‐resolution data are essential to enhance our understanding of diversity patterns and conservation efforts for freshwater biodiversity.

## Author Contributions

**Boyang Shi:** formal analysis (equal), methodology (equal), resources (equal), writing – original draft (equal), writing – review and editing (equal). **Xiyang Hao:** formal analysis (equal), resources (equal), writing – original draft (equal). **Ruxiao Wang:** resources (equal), writing – review and editing (equal). **Kelin Chu:** investigation (equal). **Da Pan:** methodology (equal), writing – review and editing (equal). **Tianyu Gu:** resources (equal). **Jun Wu:** investigation (equal). **Neil Cumberlidge:** writing – review and editing (equal). **Hongying Sun:** project administration (equal), supervision (equal), writing – original draft (equal), writing – review and editing (equal).

## Conflicts of Interest

The authors declare no conflicts of interest.

## Supporting information


Appendix S1.

Appendix S2.

Appendix S3.

Appendix S4.

Data S1.

Data S2.

Data S3.

## Data Availability

The original contributions and R code used in all analyses for this study are included in the article/Supporting Information and DRYAD repository (https://doi.org/10.5061/dryad.jsxksn0kz).
