# Supplementary material for: Underlying Spatial Diversity Patterns of Freshwater Crabs in Southern China, With Recommendations for Conservation of Freshwater Biodiversity
Source: Ecol Evol. 2025 Jun 12;15(6):e71551. doi: 10.1002/ece3.71551 (PMC12162363; doi:10.1002/ece3.71551)
Supplement: Supplementary file 1 — Appendix S1. [file ECE3-15-e71551-s006.docx]

**A****ppendix A. Table S1**–**S9**

**Table S1.** The source of the historical records of the freshwater crabs used in this study.

| Authors | Sources |
| --- | --- |
| Shi et al. 2023a | Shi BY, Pan D, Sun HY (2023a). On a new species of freshwater crab from southern China (Crustacea, Brachyura, Potamidae). Zootaxa 5383(4): 575–584. |
| Shi et al. 2023b | Shi B, Pan D, Sun H (2023b). A taxonomic revision of the freshwater crab genus *Parvuspotamon* Dai & Bo, 1994 (Decapoda, Brachyura, Potamidae): with descriptions of a new genus and two new species. ZooKeys 1183: 13. |
| Shih et al. 2023a | Shih HT, Hsu JW, Chang K, Chen MW (2023) Taxonomy and phylogeography of the freshwater crab *Geothelphusa tawu* species complex (Crustacea: Decapoda: Potamidae) from southern Taiwan and offshore islets. Zoological Studies 62. |
| Shih et al. 2023b | Shih HT, Naruse T, Schubart CD (2023). Molecular evidence and differences in gonopod morphology lead to the recognition of a new species of the freshwater crab genus *Candidiopotamon* Bott, 1967 (Crustacea, Brachyura, Potamidae) from eastern Taiwan. ZooKeys 1179: 169. |
| Lu et al. 2023 | Lu YB, Zhang YX, Zou JX (2023). The systematic position of *Cryptopotamon* *anacoluthon* (Kemp, 1918), with the description of a new species of *Sinolapotamon* Tai & Sung, 1975 (Crustacea, Decapoda, Brachyura, Potamidae) from southern China. ZooKeys 1166: 155. |
| Wang et al. 2022 | Wang RX, Pan D, Sun HY (2022). Two new species of freshwater crabs of the genera *Huananpotamon* Dai & Ng, 1994 and *Minpotamon* Dai & Türkay, 1997 (Decapoda: Brachyura: Potamidae) from eastern China. Journal of Crustacean Biology 42(2): ruac029. |
| Shi et al. 2022 | Shi BY, Pan D, Sun HY (2022). A new genus and new species of potamid crab from Yunnan, southern China (Decapoda: Brachyura: Potamidae: Potamiscinae). Journal of Crustacean Biology 42(1): ruac001. |
| Zhao et al. 2022 | Zhao JD, Xu YY, Huang C (2022). *Jianghuaimon dabiense* gen. nov. et sp. nov (Crustacea: Decapoda: Potamidae), a new genus and new species of freshwater crab from eastern-central China. Zootaxa 5168(4): 431–440. |
| Shy et al. 2021 | *Geothelphusa boreas*, a new montane freshwater crab (Crustacea: Potamidae: Geothelphusa) from northeastern Taiwan, and the identity of G. hirsuta Tan & Liu, 1998. Zootaxa 5060 (1): 93–104 |
| Huang et al. 2021 | Two new freshwater crab species of the genus *Nanhaipotamon* Bott, 1968 (Crustacea, Decapoda, Potamidae) from Huizhou, Guangdong Province, southern China. Zootaxa 5026(2): 221–238. |
| Huang et al. 2020a | Huang C, Ahyong ST, Shih HT (2020a). The second known stygomorphic freshwater crab from China, *Phasmon typhlops* gen. nov. et sp. nov. (Crustacea, Decapoda, Potamidae), diverged at the beginning of the Late Miocene. ZooKeys 1008, 1–15. |
| Huang et al. 2020b | Huang C, Huang S, Shen Z (2020b). A new long-legged terrestrial freshwater crab, *Calcipotamon puglabrum* gen. nov. et sp. nov. (Crustacea: Decapoda: Potamidae), from Hainan Island, China. *Zootaxa* 4766(3): 447–456. |
| Huang et al. 2020c | Huang C, Shih HT, Ahyong ST (2020c). The freshwater crab genus *Lacunipotamon* Dai, Song, He, Cao, Xu & Zhong, 1975 (Decapoda, Brachyura, Potamidae), with descriptions of two new species from southwestern China. Crustaceana 93(11–12): 1361–1379. |
| Huang et al. 2020d | Huang C, Wang J, Shih HT (2020d). A new genus and two new species of freshwater crab (Crustacea: Brachyura: Potamidae) with unusual coiled tip of male second gonopods from Yunnan, southwestern China. Zoological Studies 59(24): 1–14. |
| Shy et al. 2020 | Shy JY, Shih HT, Ng PKL (2020) *Crustacean Fauna of Taiwan: Brachyuran Crabs. Volume III. Freshwater Crabs - Potamidae, Gecarcinucidae*. National Penghu University of Science and Technology. |
| Zhang et al. 2020 | Zhang ZY, Pan D, Hao XY, Sun HY (2020). Two new species of freshwater crabs of the genera *Eosamon* Yeo & Ng, 2007 and *Indochinamon* Yeo & Ng, 2007 (Crustacea, Brachyura, Potamidae) from southern Yunnan, China. ZooKeys 980: 1–21. |
| Mao et al. 2020 | Mao S, Huang C (2020). Descriptions of a new species of *Minpotamon* Dai & Türkay, 1997, and a monotypic new genus of aquatic freshwater crab (Brachyura, Potamidae) from eastern Guangdong, China. Crustaceana 93(11–12): 1295–1313. |
| Wang et al. 2020a | Wang S, Xu Y, Zou JX (2020a). Description of two new species of the genus *Heterochelamon* Türkay & Dai, 1997 (Crustacea: Decapoda: Brachyura: Potamidae), from southern China. PeerJ. DOI 10.7717/peerj.9565. |
| Wang et al. 2020b | Wang S, Zhang Y, Zou JX (2020b). A new species of freshwater crab of the genus *Qianguimon* Huang, 2018 (Decapoda: Brachyura: Potamidae) from Guangxi, Southern China. PeerJ. DOI 10.7717/PeerJ*.*9194. |
| Wang et al. 2019 | Wang S, Huang C, Zou JX (2019). Description of A New Species of Freshwater Crab of the Genus *Qianguimon* Huang, 2018 (Crustacea: Decapoda: Brachyura: Potamidae) from Yulin, Guangxi, Southern China. Zoological Studies 58: e31. |
| Naruse et al. 2018 | Naruse T, Chia JE, Zhou XM (2018). Biodiversity surveys reveal eight new species of freshwater crabs (Decapoda: Brachyura: Potamidae) from Yunnan Province, China. PeerJ 6: e5497. |
| Chu et al. 2018 | Chu KL, Wang PF, Sun HY (2018). A new genus and species of primary freshwater crab and a new species of *Artopotamon* Dai & Chen, 1985 (Crustacea, Brachyura, Potamidae) from western Yunnan, China*.* Zootaxa 4422(1): 115–131. |
| Huang et al. 2018 | Huang C, Wong KC, Ahyong ST (2018a). The freshwater crabs of Macau, with the description of a new species of *Nanhaipotamon* Bott, 1968 and the redescription of *Nanhaipotamon wupingense* Cheng, Yang, Zhong & Li, 2003 (Crustacea, Decapoda, Potamidae). ZooKeys 810: 91–111. |
| Huang 2018 | Huang C (2018b). Revision of *Yarepotamon* Dai & Türkay, 1997 (Brachyura: Potamidae), freshwater crabs endemic to southern China, with descriptions of two new genera and four new species. Journal of Crustacean Biology 38(2), 173–189. |
| Huang et al. 2018 | Huang C, Shih HT, Ahyong ST (2018c). Two new genera and two new species of narrow-range freshwater crabs from Guangdong, China (Decapoda: Brachyura: Potamidae). Journal of Crustacean Biology 38(5): 614–624. |
| Ng and Win Mar 2018 | Ng PKL, Win Mar (2018). On a new species of freshwater crab, *Indochinamon* *khinpyae,* from northern Myanmar (Crustacea, Brachyura, Potamidae). ZooKeys 811: 47–63. |
| Lyu et al. 2020 | Lv YQ, Zhang ZW, Pan D, Sun HY (2020). One New record of *Indochinamon khinpyae* in China (Decapoda: Potamidae: Indochinamon). Sichuan Journal of Zoology 39(6): 671–675. |
| Huang et al. 2017 | Huang C, Ahyong ST, Shih HT (2017). *Cantopotamon*, A New Genus of Freshwater Crabs from Guangdong, China, with Descriptions of Four New Species (Crustacea: Decapoda: Brachyura: Potamidae). Zoological Studies 56: 41, 1–20. |
| Ng 2017 | Ng PKL (2017). Descriptions of two new cavernicolous species of *Chinapotamon* Dai & Naiyanetr, 1994 (Crustacea: Brachyura: Potamidae) from China. Crustacean Research 46: 1–16. |
| Do et al. 2016 | Do VT, Shih HT, Huang C (2016). A new species of freshwater crab of the genus *Tiwaripotamon* Bott, 1970 (Crustacea, Brachyura, Potamidae) from northern Vietnam and southern China. Raffles Bulletin of Zoology 64: 213–219. |
| Huang et al.2016 | Huang C, Shih H, Mao SY (2016). *Yuebeipotamon calciatile*, a new genus and new species of freshwater crab from southern China (Crustacea, Decapoda, Brachyura, Potamidae). ZooKeys 615: 61–72. |
| Huang et al. 2012 | Huang C, Huang JR, Ng PKL (2012). A new species of *Nanhaipotamon* Bott, 1968 (Crustacea: Decapoda: Brachyura: Potamidae) from Zhuhai, Guangdong Province, China*. Zootaxa 3588(1):* 55–63. |
| Zhu et al. 2010 | Zhu C, Naruse T, Zhou XM (2010). Two New Species of Freshwater Crabs of the genus *Sinolapotamon* Tai & Sung, 1975 (Decapoda, Brachyura, Potamidae) from Guangxi Zhuang autonomous region, China. Crustaceana 83(2): 245–256. |
| Yeo et al. 2007 | Yeo DCJ, Naruse T (2007). A Revision of the Freshwater Crab Genus *Hainanpotamon* Dai, 1995 (Crustacea: Decapoda: Brachyura: Potamidae: Potamiscinae), with a Redescription of *Potamon (Potamon) orientale* (Parisi, 1916) and descriptions of three New Species. Zoological Science 24(11): 1143–1158. |
| Dai 1999 | Dai AY (1999). *Fauna Sinica. Arthropoda: Crustacea: Malacostraca: Decapoda: Parathelphusidae, Potamidae*. Science Press, Beijing (In Chinese with English abstract). |
| Dai 1995 | Dai AY (1995). On a new genus and two new species of freshwater crabs from Hainan Island, China (Crustacea: Decapoda: Brachyura: Potamidae). *Acta* Zootaxonomica Sinica 20: 391–397. |
| Ng and Dudgeon 1992 | Ng PKL, Dudgeon D (1992). The Potmidae and Parathelphusidae (Crustacea: Decapoda: Brachyura) of Hong Kong. Invertebrate Systematics 6(3): 741–768. |

**Table S2.** Ecological factors in this study.

| Variable types | Ecological factors | Abbreviation | References |
| --- | --- | --- | --- |
| Bioclimate | Annual Mean Temperature | Bio1 | Stephen and Hijmans 2017 |
| Bioclimate | Mean Diurnal Range (Mean of monthly (max temp - min temp)) | Bio2 | Stephen and Hijmans 2017 |
| Bioclimate | Isothermality (Bio2/Bio7) (×100) | Bio3 | Stephen and Hijmans 2017 |
| Bioclimate | Temperature Seasonality (standard deviation ×100) | Bio4 | Stephen and Hijmans 2017 |
| Bioclimate | Max Temperature of Warmest Month | Bio5 | Stephen and Hijmans 2017 |
| Bioclimate | Min Temperature of Coldest Month | Bio6 | Stephen and Hijmans 2017 |
| Bioclimate | Temperature Annual Range (Bio5-Bio6) | Bio7 | Stephen and Hijmans 2017 |
| Bioclimate | Mean Temperature of Wettest Quarter | Bio8 | Stephen and Hijmans 2017 |
| Bioclimate | Mean Temperature of Driest Quarter | Bio9 | Stephen and Hijmans 2017 |
| Bioclimate | Mean Temperature of Warmest Quarter | Bio10 | Stephen and Hijmans 2017 |
| Bioclimate | Mean Temperature of Coldest Quarter | Bio11 | Stephen and Hijmans 2017 |
| Bioclimate | Annual Precipitation | Bio12 | Stephen and Hijmans 2017 |
| Bioclimate | Precipitation of Wettest Month | Bio13 | Stephen and Hijmans 2017 |
| Bioclimate | Precipitation of Driest Month | Bio14 | Stephen and Hijmans 2017 |
| Bioclimate | Precipitation Seasonality (Coefficient of Variation) | Bio15 | Stephen and Hijmans 2017 |
| Bioclimate | Precipitation of Wettest Quarter | Bio16 | Stephen and Hijmans 2017 |
| Bioclimate | Precipitation of Driest Quarter | Bio17 | Stephen and Hijmans 2017 |
| Bioclimate | Precipitation of Warmest Quarter | Bio18 | Stephen and Hijmans 2017 |
| Bioclimate | Precipitation of Coldest Quarter | Bio19 | Stephen and Hijmans 2017 |
| Landscape | Altitude | ALT | This study established |
| Landscape | Slope | SLO | This study established |
| Landscape | Aspect | ASP | This study established |
| Human activities | Crop | CR | Wang et al. 2015 |
| Human activities | Footprint | FP | Sanderson et al. 2002 |
| Human activities | Urban | UR | Wang et al. 2015 |
| Land cover | Forest | FR | Wang et al. 2015 |
| Land cover | Grassland | GR | Wang et al. 2015 |
| Land cover | Shrub | SH | Wang et al. 2015 |
| Land cover | Others | OT | Wang et al. 2015 |

**Table S3.** Correlation matrix of ecological variables (|*r*| < 0.75).

| Variables group | Abbreviation | ALT | ASP | CR | FR | Bio1 | Bio2 | Bio4 | Bio12 | Bio14 |
| --- | --- | --- | --- | --- | --- | --- | --- | --- | --- | --- |
| Terrain | ALT | 1.00 | -0.04 | 0.01 | -0.21 | -0.19 | 0.61 | -0.44 | -0.35 | -0.34 |
|  | ASP | -0.04 | 1.00 | -0.23 | -0.17 | 0.00 | 0.09 | -0.17 | 0.18 | 0.04 |
| Land cover | CR | -0.03 | 0.26 | 1.00 | 0.11 | 0.03 | 0.18 | 0.11 | -0.21 | -0.15 |
|  | FR | 0.05 | 0.08 | 0.03 | 1.00 | 0.04 | -0.01 | 0.05 | 0.11 | -0.02 |
| Temperature | Bio1 | -0.19 | 0.00 | 0.26 | 0.38 | 1.00 | -0.42 | -0.13 | 0.08 | 0.13 |
|  | Bio2 | 0.61 | 0.09 | 0.19 | 0.03 | -0.42 | 1.00 | -0.04 | -0.42 | -0.44 |
|  | Bio4 | -0.44 | -0.17 | 0.31 | 0.25 | -0.13 | -0.04 | 1.00 | -0.12 | 0.13 |
| Precipitation | Bio12 | -0.35 | 0.18 | -0.11 | 0.06 | 0.08 | -0.42 | -0.12 | 1.00 | 0.68 |
|  | Bio14 | -0.34 | 0.04 | 0.13 | 0.07 | 0.13 | -0.44 | 0.13 | 0.68 | 1.00 |

ALT, altitude, ASP, aspect, CR, crop, FR, forest, Bio1, annual mean temperature, Bio2, mean diurnal range (mean of monthly (max temp - min temp)), Bio4, temperature seasonality (standard deviation ×100), Bio12, annual precipitation, and Bio14, precipitation of driest month.

**Table S4.** The variance inflation factor of nine selected variables (VIF < 5).

| Variables group | Abbreviation | Variance inflation factor |
| --- | --- | --- |
| Terrain | ALT | 3.2176 |
|  | ASP | 2.8451 |
| Land cover | CR | 3.1094 |
|  | FR | 2.7958 |
| Temperature | Bio1 | 3.2564 |
|  | Bio2 | 4.1123 |
|  | Bio4 | 2.0415 |
| Precipitation | Bio12 | 2.4123 |
|  | Bio14 | 2.0015 |

ALT, altitude, ASP, aspect, CR, crop, FR, forest, Bio1, annual mean temperature, Bio2, mean diurnal range (mean of monthly (max temp - min temp)), Bio4, temperature seasonality (standard deviation ×100), Bio12, annual precipitation, and Bio14, precipitation of driest month.

**Table S5.** A checklist of freshwater crabs in the STZC. Extent of occurrence (EOO) and area of occupancy (AOO); Nine categories: Not Evaluated (NE), Extinct (EX), Extinct in the Wild (EW), Critically Endangered (CR), Endangered (EN), Vulnerable (VU), Near Threatened (NT), Least Concern (LC), and Data Deficient (DD); The arrows represent rising (**↑**) and falling (**↓**); endemic genus or species in China (*).

| **Family** | **Genus** | | **Species (Subspecies)** | | **Historical results** | **Number of sites** | **EOO**  **(km^2^)** | **AOO**  **(km^2^)** | **Assessment status** | **Status change**  **[↑, ↓ or new]** | **Assessment criteria** |
| --- | --- | --- | --- | --- | --- | --- | --- | --- | --- | --- | --- |
| Gecarcinucidae  (2 genera) | 1 | *Mekhongthelphusa* | 1 | *M. menglongensis** | NE | 1 | 4155 | 30 | NT |  |  |
|  | 2 | *Somanniathelphusa* | 2 | *S. amoyensis** | DD | 2 | 5518 | 60 | NT | new |  |
|  |  |  | 3 | *S. araeochela** | DD | 3 | 399 | 90 | NT | new |  |
|  |  |  | 4 | *S. bawangensis** | DD | 12 | 6368 | 600 | LC | new |  |
|  |  |  | 5 | *S. brevipodum** | LC | 9 | 48000 | 450 | LC |  |  |
|  |  |  | 6 | *S. chongi** | LC | 15 | 27839 | 750 | LC |  |  |
|  |  |  | 7 | *S. falx** | DD | 5 | 4404 | 250 | LC | new |  |
|  |  |  | 8 | *S. hainanensis** | DD | 10 | 5612 | 500 | LC | new |  |
|  |  |  | 9 | *S. huaanensis** | DD | 3 | 1203 | 90 | NT | new |  |
|  |  |  | 10 | *S. longicaudus** | DD | 2 | 1501 | 100 | NT | new |  |
|  |  |  | 11 | *S. megachela** | DD | 2 | 399 | 60 | NT | new |  |
|  |  |  | 12 | *S. nanningensis** | DD | 1 | 1501 | 30 | DD |  |  |
|  |  |  | 13 | *S. qiongshanensis** | DD | 10 | 5490 | 500 | LC | new |  |
|  |  |  | 14 | *S. sinensis** | DD | 3 | 5933 | 90 | NT | new |  |
|  |  |  | 15 | *S. taiwanensis** | NE | 5 | 259 | 150 | NT |  |  |
|  |  |  | 16 | *S. tongzhaensis** | DD | 15 | 9840 | 450 | LC | new |  |
|  |  |  | 17 | *S. yuilinensis** | DD | 19 | 80034 | 450 | LC | new |  |
|  |  |  | 18 | *S. zanklon** | LC | 13 | 1850 | 390 | LC |  |  |
|  |  |  | 19 | *S. zhangpuensis** | DD | 2 | 13328 | 60 | DD |  |  |
|  |  |  | 20 | *S. zhapoensis** | DD | 1 | 1364 | 30 | DD |  |  |
| Potamidae  (43 genera) | 3 | *Aiyunamon* | 21 | *A. daiae** | LC | 2 | 500 | 60 | VU | ↑ | B1ab(i,ii,iii,iv)+2ab(i,ii,iii,iv) |
|  |  |  | 22 | *A. fatum** | NE | 4 | 776 | 120 | LC |  |  |
|  |  |  | 23 | *A. lushuiense** | LC | 8 | 308 | 240 | LC |  |  |
|  |  |  | 24 | *A. tengchongense** | LC | 4 | 2816 | 120 | LC |  |  |
|  | 4 | *Aparapotamon** | 25 | *A. grahami* | LC | 50 | 511673 | 1500 | LC |  |  |
|  |  |  | 26 | *A. similium* | VU | 3 | 200 | 900 | VU |  |  |
|  | 5 | *Apotamonautes** | 27 | *A. hainanensis banshuiensis* | NE | 2 | 171 | 60 | NT |  |  |
|  |  |  | 28 | *A. hainanensis bawanglingensis* | NE | 44 | 6513 | 1320 | LC |  |  |
|  |  |  | 29 | *A. hainanensis hainanensis* | NE | 5 | 4795 | 150 | NT |  |  |
|  |  |  | 30 | *A. hainanensis nanlinensis* | NE | 5 | 824 | 150 | NT |  |  |
|  | 6 | *Barbamon** | 31 | *B. zhoui* | NE | 1 | 171 | 10 | EN |  | B1ab(i,ii,iii,iv)+2ab(i,ii,iii,iv) |
|  | 7 | *Bottapotamon** | 32 | *B. nanan* | NE | 11 | 38255 | 220 | LC |  |  |
|  | 8 | *Calcipotamon** | 33 | *C. puglabrum* | EN | 1 | 164 | 10 | EN |  | B1ab(i,ii,iii,iv)+2ab(i,ii,iii,iv) |
|  | 9 | *Candidiopotamon* | 34 | *C. rathbuni** | NE | 39 | 5714 | 1170 | LC |  |  |
|  |  |  | 35 | *C. penglai** | NE | 16 | 2857 | 480 | LC |  |  |
|  | 10 | *Cantopotamon** | 36 | *C. hengqinense* | EN | 3 | 1504 | 90 | VU | ↓ | B1ab(i,ii,iii)+2ab(i,ii,iii) |
|  |  |  | 37 | *C. shangchuanense* | NE | 1 | 1732 | 30 | VU |  | B1ab(i,ii)+2ab(i,ii) |
|  |  |  | 38 | *C. yangxiense* | NE | 1 | 601 | 30 | EN |  | B1ab(i,ii,iii,iv)+2ab(i,ii,iii,iv) |
|  |  |  | 39 | *C. zhuhaiense* | NE | 1 | 1725 | 30 | VU |  | B1ab(i,ii)+2ab(i,ii) |
|  | 11 | *Chinapotamon** | 40 | *C. clarkei* | NE | 3 | 2645 | 90 | NT |  |  |
|  |  |  | 41 | *C. dashiwei* | NE | 3 | 200 | 90 | NT |  |  |
|  |  |  | 42 | *C. depressum* | LC | 15 | 129538 | 450 | LC |  |  |
|  |  |  | 43 | *C. glabrum* | DD | 5 | 13094 | 150 | LC | new |  |
|  |  |  | 44 | *C. longlinense* | DD | 4 | 4236 | 120 | NT | new |  |
|  |  |  | 45 | *C. maolanense* | NE | 1 | 200 | 30 | VU |  | D2 |
|  |  |  | 46 | *C. pusillum* | DD | 1 | 281 | 30 | EN | new | B1ab(i,ii)+2ab(i,ii) |
|  |  |  | 47 | *C. xingrenense* | DD | 5 | 16438 | 150 | NT | new |  |
|  | 12 | *Daipotamon** | 48 | *D. minos* | LC | 2 | 4 | \ | EN | ↑ | B1ab(i,ii,iii,iv)+2ab(i,ii,iii,vi) |
|  | 13 | *Eurusamon** | 49 | *E. guangdongense* | DD | 6 | 32780 | 180 | NT | new |  |
|  | 14 | *Geothelphusa* | 50 | *G. albogilva** | LC | 19 | 1029 | 570 | LC |  |  |
|  |  |  | 51 | *G. ancylophallus** | NE | 7 | 678 | 210 | LC |  |  |
|  |  |  | 52 | *G. bicolor** | LC | 12 | 2216 | 360 | LC |  |  |
|  |  |  | 53 | *G. boreas** | NE | 1 | 507 | 30 | DD |  |  |
|  |  |  | 54 | *G. caesia** | LC | 4 | 1289 | 120 | NT | ↑ |  |
|  |  |  | 55 | *G. candidiensis** | LC | 5 | 1563 | 150 | LC |  |  |
|  |  |  | 56 | *G. chiui** | DD | 2 | 316 | 60 | VU | new | B1ab(i,ii)+2ab(i,ii) |
|  |  |  | 57 | *G. cilan** | NE | 1 | 192 | 30 | DD |  |  |
|  |  |  | 58 | *G. cinerea** | LC | 10 | 1946 | 300 | LC |  |  |
|  |  |  | 59 | *G. dolichopodes** | LC | 7 | 927 | 210 | LC |  |  |
|  |  |  | 60 | *G. eucrinodonta** | NE | 17 | 4382 | 510 | LC |  |  |
|  |  |  | 61 | *G. eurysoma** | DD | 6 | 4267 | 180 | LC | new |  |
|  |  |  | 62 | *G. ferruginea** | DD | 7 | 142 | 210 | LC | new |  |
|  |  |  | 63 | *G. gracilipes** | DD | 4 | 621 | 120 | NT | new |  |
|  |  |  | 64 | *G. haituan** | NE | 2 | 136 | 60 | VU |  | B1ab(i,ii)+2ab(i,ii) |
|  |  |  | 65 | *G. hirsuta** | NE | 4 | 1220 | 120 | NT |  |  |
|  |  |  | 66 | *G. holthuisi** | NE | 1 | 204 | 30 | DD |  |  |
|  |  |  | 67 | *G. ilan** | NE | 9 | 3016 | 270 | LC |  |  |
|  |  |  | 68 | *G. lanyu** | CR | 5 | 433 | 150 | VU | ↓ |  |
|  |  |  | 69 | *G. leeae** | DD | 1 | 396 | 30 | DD |  |  |
|  |  |  | 70 | *G. lili** | NE | 4 | 1047 | 120 | LC |  |  |
|  |  |  | 71 | *G. makatao** | NE | 4 | 285 | 120 | LC |  |  |
|  |  |  | 72 | *G. miyazakii** | NT | 10 | 674 | 300 | LC | ↓ |  |
|  |  |  | 73 | *G. monticola** | NE | 6 | 140 | 180 | LC |  |  |
|  |  |  | 74 | *G. nanao** | NE | 4 | 410 | 120 | LC |  |  |
|  |  |  | 75 | *G. nanhsi** | NE | 5 | 1151 | 150 | LC |  |  |
|  |  |  | 76 | *G. olea** | LC | 31 | 5032 | 930 | LC |  |  |
|  |  |  | 77 | *G. pingtung** | VU | 4 | 488 | 120 | VU |  |  |
|  |  |  | 78 | *G. shernshan** | NE | 3 | 420 | 90 | NT |  |  |
|  |  |  | 79 | *G. shokitai* | LC | 1 | 248 | 30 | VU | ↑ | B1ab(i,ii)+2ab(i,ii) |
|  |  |  | 80 | *G. siasiat** | NE | 2 | 871 | 60 | VU |  |  |
|  |  |  | 81 | *G. takuan** | NE | 1 | 396 | 30 | DD |  |  |
|  |  |  | 82 | *G. tali** | DD | 4 | 1079 | 120 | LC | new |  |
|  |  |  | 83 | *G. taroko** | NE | 3 | 886 | 90 | VU |  |  |
|  |  |  | 84 | *G. tawu** | DD | 11 | 2596 | 330 | LC | new |  |
|  |  |  | 85 | *G. tsayae** | NE | 5 | 2005 | 150 | LC |  |  |
|  |  |  | 86 | *G. wangi** | VU | 1 | 118 | 30 | VU |  |  |
|  |  |  | 87 | *G. wutai** | VU | 2 | 420 | 60 | VU |  |  |
|  |  |  | 88 | *G. yangmingshan** | NE | 2 | 383 | 60 | VU |  |  |
|  | 15 | *Hainanpotamon* | 89 | *H. daiae** | DD | 16 | 11596 | 480 | LC | new |  |
|  |  |  | 90 | *H. fuchengense** | DD | 4 | 904 | 120 | NT | new |  |
|  |  |  | 91 | *H. helense** | DD | 9 | 3028 | 270 | LC | new |  |
|  |  |  | 92 | *H. orientale** | EN | 8 | 5160 | 240 | NT | ↓ |  |
|  | 16 | *Heterochelamon** | 93 | *H. castanea* | NE | 2 | 138 | 60 | VU |  |  |
|  |  |  | 94 | *H. guangxiense* | DD | 1 | 489 | 30 | DD |  |  |
|  |  |  | 95 | *H. huidongense* | NE | 3 | 3028 | 90 | NT |  |  |
|  |  |  | 96 | *H. purpureomanuale* | DD | 1 | 32 | 30 | VU | new | D2 |
|  |  |  | 97 | *H. tessellatum* | NE | 4 | 692 | 120 | NT |  |  |
|  | 17 | *Huananpotamon** | 98 | *H. angulatum* | DD | 7 | 6533 | 140 | LC | new |  |
|  |  |  | 99 | *H. changzhium* | NE | 2 | 3424 | 40 | NT |  |  |
|  |  |  | 100 | *H. planopodum* | DD | 10 | 14135 | 200 | LC | new |  |
|  |  |  | 101 | *H. zhangzhouense* | DD | 6 | 588 | 120 | LC | new |  |
|  | 18 | *Indochinamon* | 102 | *I. ahkense** | NE | 2 | 201 | 60 | NT |  |  |
|  |  |  | 103 | *I. andersonianum* | DD | 2 | 1322 | 60 | NT | new |  |
|  |  |  | 104 | *I. boshanense** | LC | 4 | 14524 | 120 | LC |  |  |
|  |  |  | 105 | *I. changpoense** | DD | 3 | 3781 | 90 | NT | new |  |
|  |  |  | 106 | *I. chinghungense** | DD | 28 | 7218 | 540 | LC | new |  |
|  |  |  | 107 | *I. daweishanense** | LC | 7 | 1245 | 210 | LC |  |  |
|  |  |  | 108 | *I. edwardsi* | LC | 9 | 8786 | 280 | LC |  |  |
|  |  |  | 109 | *I. flexum** | DD | 4 | 1217 | 120 | LC | new |  |
|  |  |  | 110 | *I. gengmaense** | DD | 11 | 2613 | 330 | LC | new |  |
|  |  |  | 111 | *I. hispidum* | DD | 5 | 10967 | 150 | LC | new |  |
|  |  |  | 112 | *I. jianchuanense** | LC | 5 | 3160 | 150 | LC |  |  |
|  |  |  | 113 | *I. jinpingense* | DD | 9 | 14726 | 270 | LC | new |  |
|  |  |  | 114 | *I. khinpyae* | LC | 2 | 209 | 60 | LC |  |  |
|  |  |  | 115 | *I. lui** | LC | 18 | 20183 | 540 | LC |  |  |
|  |  |  | 116 | *I. malipoense** | NE | 6 | 2637 | 180 | LC |  |  |
|  |  |  | 117 | *I. menglaense** | NE | 3 | 7418 | 90 | LC |  |  |
|  |  |  | 118 | *I. parpidum** | NE | 2 | 1057 | 60 | NT |  |  |
|  |  |  | 119 | *I. tannanti* | DD | 10 | 24196 | 300 | LC | new |  |
|  |  |  | 120 | *I. tujiense** | LC | 4 | 8248 | 120 | LC |  |  |
|  |  |  | 121 | *I. xinpingense** | LC | 6 | 19869 | 180 | LC |  |  |
|  |  |  | 122 | *I. frontatum** | NE | 1 | 1214 | 30 | NT |  |  |
|  | 19 | *Lacunipotamon** | 123 | *L. albusorbitum* | DD | 1 | 88 | 20 | VU | new | D2 |
|  |  |  | 124 | *L. cymatile* | NE | 1 | 183 | 20 | VU |  | B1ab(i,ii,iii)+2ab(i,ii,iii) |
|  |  |  | 125 | *L. yuanshi* | NE | 4 | 247 | 80 | NT |  |  |
|  | 20 | *Luteomon** | 126 | *L. spinapodum* | NE | 1 | 558 | 20 | VU |  | B1ab(i,ii,iii)+2ab(i,ii,iii) |
|  | 21 | *Mediapotamon** | 127 | *M. angustipedum* | DD | 1 | 946 | 30 | NT | new |  |
|  | 22 | *Megapleonum** | 128 | *M. ehuangzhang* | NE | 1 | 602 | 30 | NT |  |  |
|  |  |  | 129 | *M. shenzhen* | NE | 1 | 1950 | 30 | NT |  |  |
|  | 23 | *Minpotamon** | 130 | *M. auritum* | NE | 2 | 1091 | 40 | NT |  |  |
|  |  |  | 131 | *M. kityang* | NE | 1 | 1632 | 20 | NT |  |  |
|  |  |  | 132 | *M. nasicum* | NE | 1 | 4809 | 20 | NT |  |  |
|  | 24 | *Minutomon** | 133 | *M. shanweiense* | NE | 3 | 1514 | 60 | NT |  |  |
|  | 25 | *Nanhaipotamon** | 134 | *N. aculatum* | DD | 2 | 3782 | 60 | NT | new |  |
|  |  |  | 135 | *N. aureomarginatum* | NE | 3 | 3231 | 90 | LC |  |  |
|  |  |  | 136 | *N. formosanum* | VU | 19 | 6490 | 570 | LC | ↓ |  |
|  |  |  | 137 | *N. guangdongense* | LC | 1 | 5326 | 30 | DD |  |  |
|  |  |  | 138 | *N. hongkongense* | LC | 3 | 4666 | 90 | NT | ↑ |  |
|  |  |  | 139 | *N. huaanense* | DD | 2 | 588 | 60 | NT | new |  |
|  |  |  | 140 | *N. incendium* | NE | 2 | 1681 | 60 | NT |  |  |
|  |  |  | 141 | *N. longhaiense* | NE | 3 | 497 | 90 | LC |  |  |
|  |  |  | 142 | *N. macau* | NT | 1 | 1504 | 30 | EN | ↑ |  |
|  |  |  | 143 | *N. nanriense* | DD | 8 | 4635 | 240 | NT | new |  |
|  |  |  | 144 | *N. pingtanense* | NE | 2 | 713 | 60 | NT |  |  |
|  |  |  | 145 | *N. pingyuanense* | DD | 3 | 1120 | 90 | LC | new |  |
|  |  |  | 146 | *N. yongchuense* | DD | 3 | 4491 | 90 | LC | new |  |
|  |  |  | 147 | *N. zhuhaiense* | NE | 2 | 1505 | 60 | NT |  |  |
|  | 26 | *Neotiwaripotamon** | 148 | *N. jianfengense* | DD | 15 | 4635 | 450 | LC | new |  |
|  |  |  | 149 | *N. whiteheadi* | DD | 5 | 1810 | 150 | NT | new |  |
|  | 27 | *Parapotamon** | 150 | *P. spinescens* | VU | 4 | 30918 | 160 | LC | ↓ |  |
|  | 28 | *Pararanguna** | 151 | *P. hemicyclia* | EN | 2 | 940 | 40 | EN |  | B1ab(i,ii,iii,iv)+2ab(i,ii,iii) |
|  |  |  | 152 | *P. semilunatum* | LC | 2 | 3000 | 40 | VU | ↑ | D2 |
|  | 29 | *Paratelphusula* | 153 | *P. burmensis* | DD | 1 | 359 | 30 | LC | new |  |
|  | 30 | *Parvuspotamon** | 154 | *P. yuxiense* | VU | 2 | 539 | 60 | VU |  | B1ab(i,ii)+2ab(i,ii,iii) |
|  | 31 | *Phasmon** | 155 | *P. typhlops* | NE | 1 | 1190 | 30 | EN |  | B1ab(i,ii,iii,iv)+2ab(i,ii,iii) |
|  | 32 | *Potamiscus* | 156 | *P. cangyuanensis** | DD | 1 | 766 | 30 | VU | new | B1ab(i,ii)+2ab(i,ii,iii) |
|  |  |  | 157 | *P. montosus** | DD | 3 | 1593 | 90 | NT | new |  |
|  |  |  | 158 | *P. yiwuensis** | DD | 3 | 1264 | 90 | NT | new |  |
|  | 33 | *Pusillamon** | 159 | *P. baishuiense* | DD | 2 | 260 | 20 | VU | new | D2 |
|  |  |  | 160 | *P. huaningense* | DD | 2 | 230 | 20 | VU | new | D2 |
|  |  |  | 161 | *P. panxiense* | DD | 1 | 27 | 10 | VU | new | D2 |
|  |  |  | 162 | *P. tonghaiense* | DD | 3 | 875 | 30 | NT | new |  |
|  |  |  | 163 | *P. xinpingense* | DD | 2 | 235 | 20 | VU | new | B1ab(i,ii,iii)+2ab(i,ii,iii) |
|  |  |  | 164 | *P. yuxiense* | DD | 2 | 2415 | 20 | NT | new |  |
|  | 34 | *Qianguimon** | 165 | *Q. aflagellum* | DD | 4 | 240 | 80 | LC | new |  |
|  |  |  | 166 | *Q. rongxianense* | NE | 1 | 1099 | 20 | DD | ↓ |  |
|  |  |  | 167 | *Q. splendidum* | NE | 1 | 138 | 20 | DD | ↓ |  |
|  |  |  | 168 | *Q. yuzhouense* | NE | 1 | 877 | 20 | DD | ↓ |  |
|  | 35 | *Semicirculara** | 169 | *S. lincangensis* | NE | 4 | 2926 | 120 | NT |  |  |
|  | 36 | *Sinolapotamon** | 170 | *S. auriculatum* | NE | 3 | 1246 | 20 | NT |  |  |
|  |  |  | 171 | *S. anacoluthon* | VU | 1 | 50 | 20 | VU |  | B1ab(i,ii,iii)+2ab(i,ii,iii) |
|  |  |  | 172 | *S. cirratum* | NE | 2 | 1117 | 60 | NT |  |  |
|  |  |  | 173 | *S. palmatum* | DD | 3 | 1074 | 90 | NT | new |  |
|  |  |  | 174 | *S. patellifer* | LC | 5 | 11286 | 150 | LC |  |  |
|  | 37 | *Sinopotamon** | 175 | *S. anyuanense* | LC | 15 | 20683 | 450 | LC |  |  |
|  |  |  | 176 | *S. exiguum* | LC | 23 | 37 | 690 | LC |  |  |
|  |  |  | 177 | *S. fukienense* | LC | 60 | 2582 | 1800 | LC |  |  |
|  |  |  | 178 | *S. jianglense* | LC | 12 | 9094 | 360 | LC |  |  |
|  |  |  | 179 | *S. longlinense* | DD | 3 | 179 | 90 | LC | new |  |
|  |  |  | 180 | *S. pinheensis* | NE | 4 | 9439 | 120 | LC |  |  |
|  |  |  | 181 | *S. rongshuiense* | LC | 9 | 14744 | 270 | LC |  |  |
|  |  |  | 182 | *S. zhangzhouense* | NE | 2 | 1082 | 60 | NT |  |  |
|  | 38 | *Songpotamon** | 183 | *S. funingense* | NE | 2 | 532 | 60 | NT |  |  |
|  |  |  | 184 | *S. malipoense* | NE | 1 | 476 | 30 | VU |  | B1ab(i,ii)+2ab(i,ii,iii) |
|  |  |  | 185 | *S. dixuense* | NT | 8 | 1904 | 240 | LC | ↑ |  |
|  | 39 | *Tenuipotamon** | 186 | *T. purpura* | DD | 2 | 1316 | 40 | EN | new | B1ab(i,ii,iii,iv)+2ab(i,ii,iii,iv) |
|  | 40 | *Teoswamon** | 187 | *T. scolasticum* | NE | 3 | 1278 | 60 | VU |  | B1ab(i,ii,iii)+2ab(i,ii,iii) |
|  | 41 | *Teretamon* | 188 | *T. husicum** | NE | 1 | 1213 | 20 | EN |  | B1ab(i,ii,iii,iv)+2ab(i,ii,iii,iv) |
|  | 42 | *Tiwaripotamon* | 189 | *T. pingguoense** | DD | 1 | 1543 | 30 | NT | new |  |
|  |  |  | 190 | *T. pluviosum* | DD | 2 | 1477 | 60 | NT | new |  |
|  | 43 | *Tortomon* | 191 | *T. gejiu** | NE | 3 | 495 | 90 | NT |  |  |
|  |  |  | 192 | *T. puer** | NE | 1 | 437 | 30 | EN |  | B1ab(i,ii,iii,iv)+2ab(iii,iv) |
|  | 44 | *Trichopotamon** | 193 | *T. daliense* | EN | 3 | 12 | 90 | VU |  | B1ab(i,ii)+2ab(i,ii,iii) |
|  | 45 | *Yarepotamon** | 194 | *Y. breviflagellum* | DD | 6 | 13281 | 180 | LC | new |  |
|  |  |  | 195 | *Y. gracillipa* | DD | 3 | 102 | 90 | LC | new |  |

**Table S6.** Summary of species in each diversity hotspot (* and ** indicate Vulnerable (VU) and Endangered (EN) status, respectively). Hotspot 1, Yongde Daisetsuzan and the surrounding area (YDSA); Hotspot 2, Wuliang Mountains and Ailao Mountains region (WAM); Hotspot 3, Dai Autonomous Prefecture of Xishuangbanna (DAPX); Hotspot 4, southern Ailao Mountains and lower reaches of Yuanjiang River (SAMY); Hotspot 5, County-Maguan and County-Malipo County in Wenshan Prefecture(MMWP); Hotspot 6, Wuyishan Mountains (WYS); Hotspot 7, karst areas in Guangxi and Guizhou (KAGG); Hotspot 8, Pearl River Delta, Hong Kong, and Macau (PHM); Hotspot 9, southwestern part of Hainan Island (HI); Hotspot 10 and 11, northwestern and southern terminus of Central Mountains in Taiwan (NCMT and SCMT, respectively)

| Hotspots | *genera* | *Species* |
| --- | --- | --- |
| 1 (YDSA) | *Aiyunamon* | *A. fatum* |
|  | *Indochinamon* | *I. hispidum; I. lui; I. gengmaense; I. edwardsii* |
|  | *Parapotamon* | *P. spinescens* |
|  | *Pararanguna* | *P. hemicyclia *; P. semilunata* |
|  | *Potamiscus* | *P. cangyuanensis ** |
|  | *Semicirculara* | *S. lincangensis* |
|  | *Somanniathelphusa* | *S. chongi* |
| 2 (WAM) | *Aparapotamon* | *A. similium ** |
|  | *Indochinamon* | *I. boshanense; I. xinpingense; I. lui; I. daweishanense; I. tujiense; I. parpidum* |
|  | *Parapotamon* | *P. spinescens* |
|  | *Parvuspotamon* | *P. yuxiense* |
|  | *Pusillamon* | *P. yuxiense; P. tonghaiense; P. panxiense *; P. xingpingense *; P. huaningense *; P. baishuiense ** |
|  | *Somanniathelphusa* | *S. chongi; S. brevipodum* |
| 3 (DAPX) | *Indochinamon* | *I. chinghungense; I. hispidum; I. daweishanense; I. menglaense; I. lui* |
|  | *Tenuipotamon* | *T. purpura *** |
|  | *Somanniathelphusa* | *S. chongi; S. brevipodum* |
|  | *Potamiscus* | *P. montosus; P. yiwuensis* |
|  | *Mekhongthelphusa* | *M. menglongensis* |
|  | *Tortomon* | *T. puer *** |
| 4 (SAMY) | *Indochinamon* | *I. parpidum; I. chinghungense; I. tannanti; I. jinpingense; I. changpoense; I. daweishanense* |
|  | *Parapotamon* | *P. spinescens* |
|  | *Somanniathelphusa* | *S. brevipodum* |
|  | *Tortomon* | *T. gejiu* |
| 5 (MMWP) | *Indochinamon* | *I. tannanti; I. malipoense* |
|  | *Barbamon* | *B. zhoui *** |
|  | *Lacunipotamon* | *L. yuanshi* |
|  | *Chinapotamon* | *C. depressum* |
|  | *Somanniathelphusa* | 1. *brevipodum* |
| 6 (WYS) | *Bottpotamon* | *B. nanan* |
|  | *Huananpotamon* | *H. planopodum* |
|  | *Minpotamon* | *M. auritum; M. nasicum* |
|  | *Sinopotamon* | *S. jianglense; S. zhangzhouense; S. pinheensis* |
|  | *Nanhaipotamon* | *N. yongchuense; N. huaanense; N. longhaiense* |
| 7 (KAGG) | *Chinapotamon* | 1. *glabrum; C. charkei; C. depressum; C. dashiwei; C. anlongense; C. pusillum *** |
|  | *Heterochelamon* | *H. guangxiense; H. tessellatum; H. castanea ** |
|  | *Indochinamon* | *I. ahkense* |
|  | *Qianguimon* | *Q. aflagellum; Q. splendidum* |
|  | *Sinolapotamon* | *S. palmatum; S. auriculatum* |
|  | *Somanniathelphusa* | *S. longicaudus S. araeochela* |
| 8 (PHM) | *Cantopotamon* | *C. zhuhaiense; C. hengqinense ** |
|  | *Sinolapotamon* | *S. anacoluthon* |
|  | *Eurusamon* | *E. guangdongense* |
|  | *Nanhaipotamon* | *N. aculatum; N. hongkongense; N. zhuhaiense; N. macau *** |
|  | *Megapleonum* | *M. shenzhen* |
|  | *Somanniathelphusa* | *S. zanklon; S. anacoluthon ** |
| 9 (HI) | *Hainanpotamon* | *H. daiae; H. fuchengense; H. helense; H. orientale* |
|  | *Apotamonautes* | *A. hainanensis banshuiensis; A. hainanensis bawanglingensis; A. hainanensis hainanensis; A. hainanensis nanlinensis* |
|  | *Somanniathelphusa* | *S. banwangensis; S. hainanensis; S. tongzhaensis* |
|  | *Neotiwaripotamon* | *N. jianfengense; N. whiteheadi* |
|  | *Calcipotamon* | *C. puglabrum *** |
| 10 (SCMT) | *Geothelphusa* | *G. olea; G. takuan; G. hirsuta; G. eucrinodonta; G. caesia; G. siasiat ** |
|  | *Candidiopotamon* | *C. rathbuni* |
| 11 (NCMT) | *Geothelphusa* | *G. ancylophallus; G. albogilva; G. caesia; G. pingtung *; G. lili; G. wutai *; G. shernshan; G. olea; G. tsayae; G. tawu; G. lanyu *; G. ferruginea; G. holthuisi; G. makatao; G. nanhsi* |
|  | *Candidiopotamon* | *C. rathbuni,* |
|  | *Nanhaipotamon* | *N. formosanum* |
|  | *Somanniathelphusa* | *S. taiwanensis* |

**Table S7.** Descriptive statistics of MLR between richness and ecological variables.

|  | Estimate | Standard error | Statistic | *p* value |
| --- | --- | --- | --- | --- |
| ALT | 0.001 | 0.000 | 4.216 | *** |
| CR | 1.112 | 0.002 | -3.442 | *** |
| FR | 1.089 | 0.003 | 3.214 | *** |
| BIO1 | -0.010 | 0.001 | 2.234 | ** |
| BIO14 | 0.015 | 0.012 | -2.987 | ** |

ALT, altitude, CR, crop, FR, forest, Bio1, annual mean temperature, and Bio14, precipitation of driest month. Signif. codes: *** *p* < 0.001, ** *p* < 0.01; Residual standard error: 0.5393 on 330 degrees of freedom; Multiple R^2^ = 0.355, Adjusted R^2^ = 0.347; F-statistic = 45.36 on 330 degrees of freedom; *p* value < 0.001.

**Table S8.** Diagnostic Measure for multiple linear regression (MLR), and geographically weighted regression (GWR).

| Models | R^2^ | Adjusted R^2^ | AIC | AICc | RSS |
| --- | --- | --- | --- | --- | --- |
| GWR | 0.730 | 0.671 | 740.530 | 784.750 | 424.765 |
| MLR | 0.481 | 0.467 | 853.292 | 856.910 | 817.044 |

AIC, Akaike information criteria, AICc, Akaike Information Criterion corrected, and RSS, Residual Sum of Squares.

**Table S9.** Descriptive statistics of geographically weighted regression between richness and ecological variables.

|  | Minimum | 25% quartile | Median | 75% quartile | Maximum |
| --- | --- | --- | --- | --- | --- |
| ALT | -3.007E+01 | -1.615E+00 | 7.428E+00 | 1.384E+01 | 2.039E+01 |
| CR | -6.614E-03 | -1.304E-03 | -3.832E-04 | 3.384E-03 | 8.800E-03 |
| FR | -1.841E+00 | -9.642E-01 | -2.158E-01 | 6.663E-01 | 3.957E+00 |
| BIO1 | -1.170E-02 | -2.461E-03 | -8.913E-04 | 1.230E-03 | 9.900E-03 |
| BIO14 | -1.211E+00 | -2.122E-03 | -1.318E-01 | 5.221E-01 | 3.213E+01 |

ALT, altitude, CR, crop, FR, forest, Bio1, annual mean temperature, and Bio14, precipitation of driest month.
