# Supplementary material for: Underlying Spatial Diversity Patterns of Freshwater Crabs in Southern China, With Recommendations for Conservation of Freshwater Biodiversity
Source: Ecol Evol. 2025 Jun 12;15(6):e71551. doi: 10.1002/ece3.71551 (PMC12162363; doi:10.1002/ece3.71551)
Supplement: Supplementary file 2 — Appendix S2. [file ECE3-15-e71551-s004.docx]

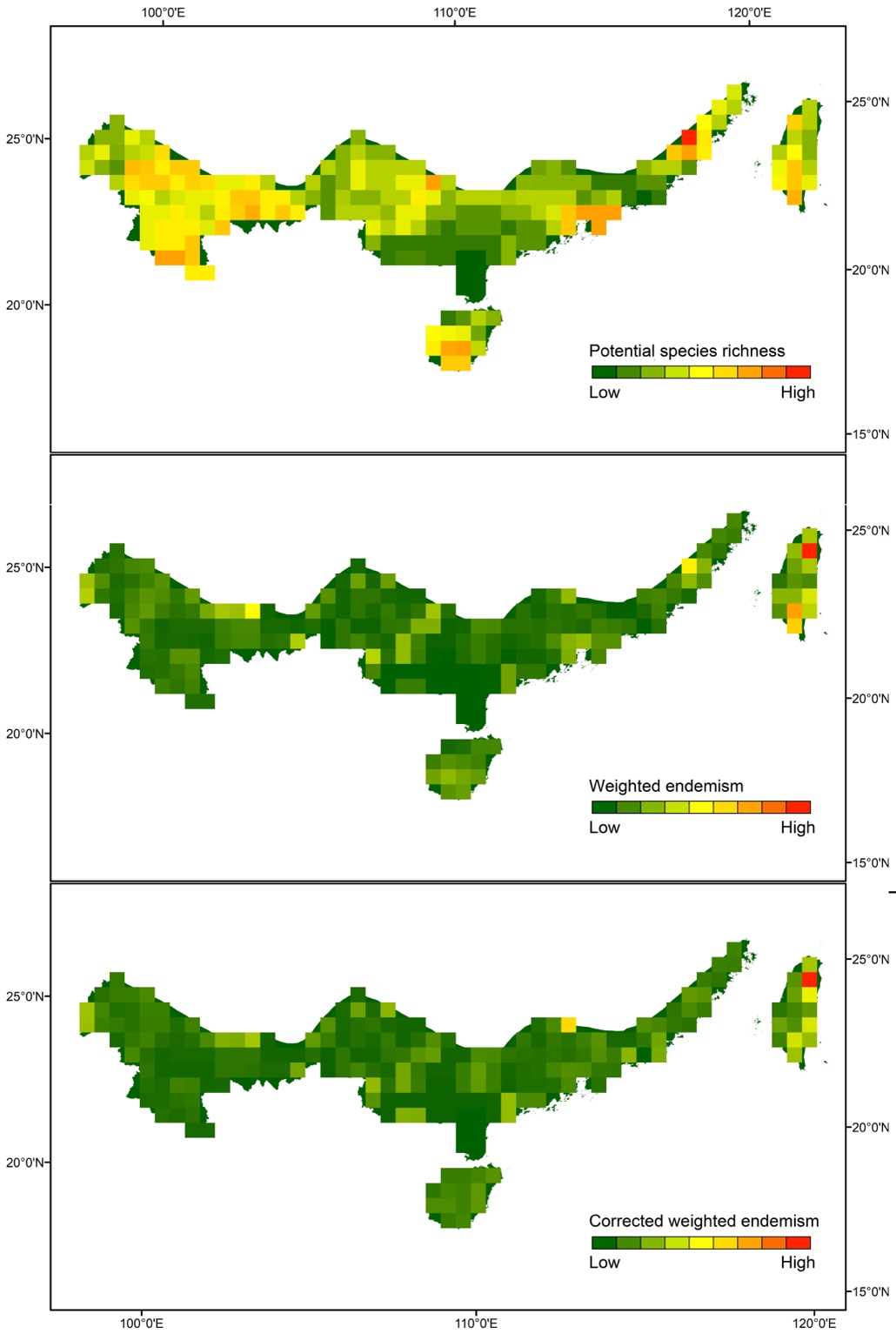
**Appendix B. Figure S1 and S2**

**Fig. S1.** Potential richness pattern, weighted endemism, and corrected weighted endemism of freshwater crabs based on 0.5° grids (about 50 km).


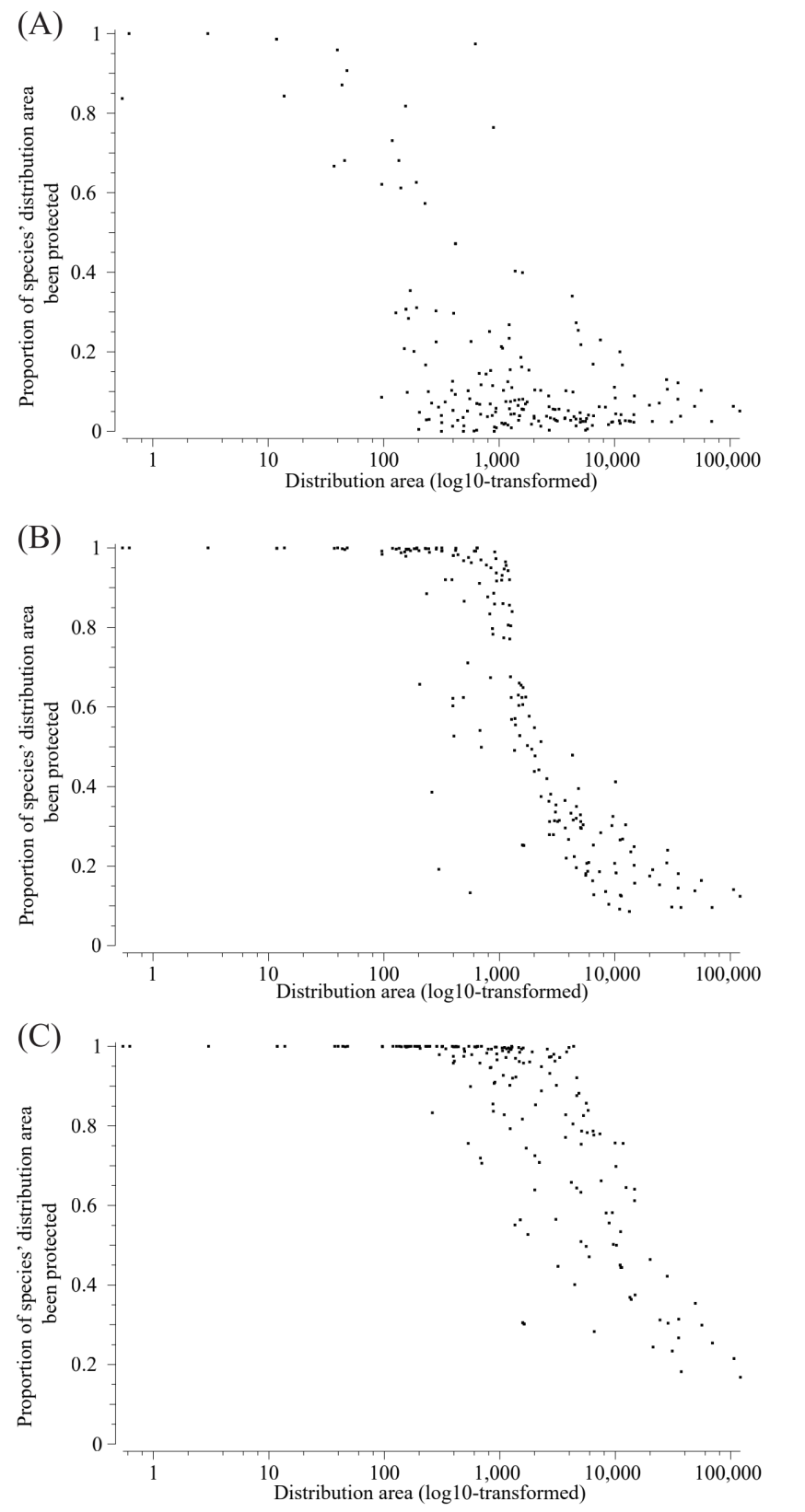


**Fig. S2.** The proportion of each species distribution range protected by current NRs (A: 5.7% of the region) and potential conservation priority areas (B: 15% of the region; C: 30% of the region).
