# Supplementary material for: Underlying Spatial Diversity Patterns of Freshwater Crabs in Southern China, With Recommendations for Conservation of Freshwater Biodiversity
Source: Ecol Evol. 2025 Jun 12;15(6):e71551. doi: 10.1002/ece3.71551 (PMC12162363; doi:10.1002/ece3.71551)
Supplement: Supplementary file 3 — Appendix S3. [file ECE3-15-e71551-s002.docx]

**Appendix C.**

**The details of freshwater crab species in hotspot 1-11.**

Hotspot 1 (YDSA) is home to seven genera and 11 species, including *Aiyunamon* (1 species), *Indochinamon* (4 species), *Parapotamon* (1 species), *Pararanguna* (2 species), *Potamiscus* (1 species), *Semicirculara* (1 species), and *Somanniathelphusa* (1 species). Species in this region are primarily found at high elevations, often residing beneath rocks in mountain streams. This area is also the most ecologically complex region in the Hengduan Mountains, where altitude variations and dry-hot river valleys contribute to its unique landscape, fostering species diversity and endemism. While altitude habitats may offer a refuge, it also carries the risk of isolating populations.

Hotspot 2 (WAM) is distributed along the two longitudinal mountain ranges, Wuliang Mountains and Ailao Mountains, exhibiting ribbon-like distributions. Six genera and 17 species are found in this area, including *Aparapotamon* (1 species), *Indochinamon* (6 species), *Parapotamon* (1 species), *Parvuspotamon* (1 species), *Pusillamon* (6 species), *Somanniathelphusa* (2 species). Species in this region exhibit significant morphological and habitat differences. For example, *Indochinamon*, *Parapotamon*, and *Somanniathelphusa* are larger and often found under rocks in mountain streams, while *Parvuspotamon*, *Aparapotamon*, and *Pusillamon*, which have smaller carapaces, typically burrow along streambanks. In addition, inhabiting in lowland streams and lakes, *Parapotamon* and *Somanniathelphusa* have developed sharp epibranchial teeth, which help protect them from predation.

Hotspot 3 (DAPX) encompasses most of the Dai Autonomous Prefecture of Xishuangbanna. It harbors six genera of ten species, belonging to *Indochinamon* (5 species), *Tenuipotamon* (1 species), *Somanniathelphusa* (2 species), and *Potamiscus* (2 species)*, Mekhongthelphusa* (1 species), and *Tortomon* (1 species)*.* The region is most unique to *Tenuipotamon*, as its distribution is dominated by burrowing on the banks of small stream in the high mountains and has smallest body size than other freshwater crabs (carapace width of the smallest mature individual = 8 mm).

Hotspot 4 (SAMY), situated in the southern Ailao Mountains and lower reaches of Yuanjiang River, includes four genera of nine stream/lake-inhabiting species: *Indochinamon* (6 species), *Parapotamon* (1 species), *Tortomon* (1 species), and *Somanniathelphusa* (1 species). Hotspot 5 (MMWP), located in County-Maguan and County-Malipo County in Wenshan Prefecture, comprises five genera of six species including *Indochinamon* (2 species), *Barbamon* (1 species)*, Lacunipotamon* (1 species)*, Chinapotamon* (1 species), and *Somanniathelphusa* (1 species).

Hotspot 6 (WYS) is located in the Wuyishan Mountains, where ten species in five genera are distributed, including *Bottpotamon* (1 species), *Huananpotamon* (1 species), *Minpotamon* (2 species), *Nanhaipotamon* (3 species), and *Sinopotamon* (3 species). The first three genera are smaller in carapace-size and are found under debris in streams or in burrows along banks. The carapace of *Sinopotamon* and *Nanhaipotamon* are relatively larger but occupy very different habitats: *Sinopotamon* seeks shelter under rocks in streams, while *Nanhaipotamon* burrows in mud along the banks.

Hotspot 7 (KAGG) is characterized by unique karst areas in Guangxi and Guizhou, where 13 species in six genera are distributed, including *Chinapotamon* (6 species), *Heterochelamon* (3 species), *Indochinamon* (1 species), *Qianguimon* (2 species), *Sinolapotamon* (2 species), and *Somanniathelphusa* (2 species). Several species of *Chinapotamon* have been found in the underground rivers of karst caves, while others inhabit mountain streams.

Hotspot 8 (PHM) located in Pearl River Delta, Hong Kong, and Macau, containing *Cantopotamon* (2 species), *Sinolapotamon* (1 species), *Eurusamon* (1 species), *Nanhaipotamon* (4 species), *Megapleonum* (1 species), and *Somanniathelphusa* (2 species).

Hotspot 9 (HI) on southwestern part of Hainan Island encompasses five genera and 11 species, including burrowing (*Hainanpotamon*, 4 species), inhabiting in lowland stream (*Apotamonautes*, 1 species and 4 subspecies, and *Somanniathelphusa*, 3 species), and hiding in the water-filled crevices of tree (*Neotiwaripotamon*, 2 species) and limestone (*Calcipotamon*, 1 species).

Hotspots 10 and 11 on Taiwan Island are predominantly distributed in the northwestern and southern terminus of the Central Mountains in Taiwan (NCMT and SCMT, respectively). Hotspot 10 includes two genera with seven species, namely *Geothelphusa* (6 species) and *Candidiopotamon* (1 species). Meanwhile, Hotspot 11 comprises four genera of 18 species, including *Geothelphusa* (15 species), *Candidiopotamon* (1 species), *Nanhaipotamon* (1 species) and *Somanniathelphusa* (1 species). *Geothelphusa* species inhabit montane streams with relatively large elevation differences in habitat between species. *Somanniathelphusa* is predominantly found in canals next to lowland rice paddies, while *Nanhaipotamon* typically burrows along the stream banks. The *Candidiopotamon* species are primarily distributed on both sides of the Central Mountain Range, with a wide range of distribution.
