# Supplementary material for: Underlying Spatial Diversity Patterns of Freshwater Crabs in Southern China, With Recommendations for Conservation of Freshwater Biodiversity
Source: Ecol Evol. 2025 Jun 12;15(6):e71551. doi: 10.1002/ece3.71551 (PMC12162363; doi:10.1002/ece3.71551)
Supplement: Supplementary file 4 — Appendix S4. [file ECE3-15-e71551-s003.docx]

# Supporting information

**A****ppendix A. Table S1**–**S9**

**Table S1.** The source of the historical records of the freshwater crabs used in this study.

| Authors | Sources |
| --- | --- |
| Shi et al. 2023a | Shi BY, Pan D, Sun HY (2023a). On a new species of freshwater crab from southern China (Crustacea, Brachyura, Potamidae). Zootaxa 5383(4): 575–584. |
| Shi et al. 2023b | Shi B, Pan D, Sun H (2023b). A taxonomic revision of the freshwater crab genus *Parvuspotamon* Dai & Bo, 1994 (Decapoda, Brachyura, Potamidae): with descriptions of a new genus and two new species. ZooKeys 1183: 13. |
| Shih et al. 2023a | Shih HT, Hsu JW, Chang K, Chen MW (2023) Taxonomy and phylogeography of the freshwater crab *Geothelphusa tawu* species complex (Crustacea: Decapoda: Potamidae) from southern Taiwan and offshore islets. Zoological Studies 62. |
| Shih et al. 2023b | Shih HT, Naruse T, Schubart CD (2023). Molecular evidence and differences in gonopod morphology lead to the recognition of a new species of the freshwater crab genus *Candidiopotamon* Bott, 1967 (Crustacea, Brachyura, Potamidae) from eastern Taiwan. ZooKeys 1179: 169. |
| Lu et al. 2023 | Lu YB, Zhang YX, Zou JX (2023). The systematic position of *Cryptopotamon* *anacoluthon* (Kemp, 1918), with the description of a new species of *Sinolapotamon* Tai & Sung, 1975 (Crustacea, Decapoda, Brachyura, Potamidae) from southern China. ZooKeys 1166: 155. |
| Wang et al. 2022 | Wang RX, Pan D, Sun HY (2022). Two new species of freshwater crabs of the genera *Huananpotamon* Dai & Ng, 1994 and *Minpotamon* Dai & Türkay, 1997 (Decapoda: Brachyura: Potamidae) from eastern China. Journal of Crustacean Biology 42(2): ruac029. |
| Shi et al. 2022 | Shi BY, Pan D, Sun HY (2022). A new genus and new species of potamid crab from Yunnan, southern China (Decapoda: Brachyura: Potamidae: Potamiscinae). Journal of Crustacean Biology 42(1): ruac001. |
| Zhao et al. 2022 | Zhao JD, Xu YY, Huang C (2022). *Jianghuaimon dabiense* gen. nov. et sp. nov (Crustacea: Decapoda: Potamidae), a new genus and new species of freshwater crab from eastern-central China. Zootaxa 5168(4): 431–440. |
| Shy et al. 2021 | *Geothelphusa boreas*, a new montane freshwater crab (Crustacea: Potamidae: Geothelphusa) from northeastern Taiwan, and the identity of G. hirsuta Tan & Liu, 1998. Zootaxa 5060 (1): 93–104 |
| Huang et al. 2021 | Two new freshwater crab species of the genus *Nanhaipotamon* Bott, 1968 (Crustacea, Decapoda, Potamidae) from Huizhou, Guangdong Province, southern China. Zootaxa 5026(2): 221–238. |
| Huang et al. 2020a | Huang C, Ahyong ST, Shih HT (2020a). The second known stygomorphic freshwater crab from China, *Phasmon typhlops* gen. nov. et sp. nov. (Crustacea, Decapoda, Potamidae), diverged at the beginning of the Late Miocene. ZooKeys 1008, 1–15. |
| Huang et al. 2020b | Huang C, Huang S, Shen Z (2020b). A new long-legged terrestrial freshwater crab, *Calcipotamon puglabrum* gen. nov. et sp. nov. (Crustacea: Decapoda: Potamidae), from Hainan Island, China. *Zootaxa* 4766(3): 447–456. |
| Huang et al. 2020c | Huang C, Shih HT, Ahyong ST (2020c). The freshwater crab genus *Lacunipotamon* Dai, Song, He, Cao, Xu & Zhong, 1975 (Decapoda, Brachyura, Potamidae), with descriptions of two new species from southwestern China. Crustaceana 93(11–12): 1361–1379. |
| Huang et al. 2020d | Huang C, Wang J, Shih HT (2020d). A new genus and two new species of freshwater crab (Crustacea: Brachyura: Potamidae) with unusual coiled tip of male second gonopods from Yunnan, southwestern China. Zoological Studies 59(24): 1–14. |
| Shy et al. 2020 | Shy JY, Shih HT, Ng PKL (2020) *Crustacean Fauna of Taiwan: Brachyuran Crabs. Volume III. Freshwater Crabs - Potamidae, Gecarcinucidae*. National Penghu University of Science and Technology. |
| Zhang et al. 2020 | Zhang ZY, Pan D, Hao XY, Sun HY (2020). Two new species of freshwater crabs of the genera *Eosamon* Yeo & Ng, 2007 and *Indochinamon* Yeo & Ng, 2007 (Crustacea, Brachyura, Potamidae) from southern Yunnan, China. ZooKeys 980: 1–21. |
| Mao et al. 2020 | Mao S, Huang C (2020). Descriptions of a new species of *Minpotamon* Dai & Türkay, 1997, and a monotypic new genus of aquatic freshwater crab (Brachyura, Potamidae) from eastern Guangdong, China. Crustaceana 93(11–12): 1295–1313. |
| Wang et al. 2020a | Wang S, Xu Y, Zou JX (2020a). Description of two new species of the genus *Heterochelamon* Türkay & Dai, 1997 (Crustacea: Decapoda: Brachyura: Potamidae), from southern China. PeerJ. DOI 10.7717/peerj.9565. |
| Wang et al. 2020b | Wang S, Zhang Y, Zou JX (2020b). A new species of freshwater crab of the genus *Qianguimon* Huang, 2018 (Decapoda: Brachyura: Potamidae) from Guangxi, Southern China. PeerJ. DOI 10.7717/PeerJ*.*9194. |
| Wang et al. 2019 | Wang S, Huang C, Zou JX (2019). Description of A New Species of Freshwater Crab of the Genus *Qianguimon* Huang, 2018 (Crustacea: Decapoda: Brachyura: Potamidae) from Yulin, Guangxi, Southern China. Zoological Studies 58: e31. |
| Naruse et al. 2018 | Naruse T, Chia JE, Zhou XM (2018). Biodiversity surveys reveal eight new species of freshwater crabs (Decapoda: Brachyura: Potamidae) from Yunnan Province, China. PeerJ 6: e5497. |
| Chu et al. 2018 | Chu KL, Wang PF, Sun HY (2018). A new genus and species of primary freshwater crab and a new species of *Artopotamon* Dai & Chen, 1985 (Crustacea, Brachyura, Potamidae) from western Yunnan, China*.* Zootaxa 4422(1): 115–131. |
| Huang et al. 2018 | Huang C, Wong KC, Ahyong ST (2018a). The freshwater crabs of Macau, with the description of a new species of *Nanhaipotamon* Bott, 1968 and the redescription of *Nanhaipotamon wupingense* Cheng, Yang, Zhong & Li, 2003 (Crustacea, Decapoda, Potamidae). ZooKeys 810: 91–111. |
| Huang 2018 | Huang C (2018b). Revision of *Yarepotamon* Dai & Türkay, 1997 (Brachyura: Potamidae), freshwater crabs endemic to southern China, with descriptions of two new genera and four new species. Journal of Crustacean Biology 38(2), 173–189. |
| Huang et al. 2018 | Huang C, Shih HT, Ahyong ST (2018c). Two new genera and two new species of narrow-range freshwater crabs from Guangdong, China (Decapoda: Brachyura: Potamidae). Journal of Crustacean Biology 38(5): 614–624. |
| Ng and Win Mar 2018 | Ng PKL, Win Mar (2018). On a new species of freshwater crab, *Indochinamon* *khinpyae,* from northern Myanmar (Crustacea, Brachyura, Potamidae). ZooKeys 811: 47–63. |
| Lyu et al. 2020 | Lv YQ, Zhang ZW, Pan D, Sun HY (2020). One New record of *Indochinamon khinpyae* in China (Decapoda: Potamidae: Indochinamon). Sichuan Journal of Zoology 39(6): 671–675. |
| Huang et al. 2017 | Huang C, Ahyong ST, Shih HT (2017). *Cantopotamon*, A New Genus of Freshwater Crabs from Guangdong, China, with Descriptions of Four New Species (Crustacea: Decapoda: Brachyura: Potamidae). Zoological Studies 56: 41, 1–20. |
| Ng 2017 | Ng PKL (2017). Descriptions of two new cavernicolous species of *Chinapotamon* Dai & Naiyanetr, 1994 (Crustacea: Brachyura: Potamidae) from China. Crustacean Research 46: 1–16. |
| Do et al. 2016 | Do VT, Shih HT, Huang C (2016). A new species of freshwater crab of the genus *Tiwaripotamon* Bott, 1970 (Crustacea, Brachyura, Potamidae) from northern Vietnam and southern China. Raffles Bulletin of Zoology 64: 213–219. |
| Huang et al.2016 | Huang C, Shih H, Mao SY (2016). *Yuebeipotamon calciatile*, a new genus and new species of freshwater crab from southern China (Crustacea, Decapoda, Brachyura, Potamidae). ZooKeys 615: 61–72. |
| Huang et al. 2012 | Huang C, Huang JR, Ng PKL (2012). A new species of *Nanhaipotamon* Bott, 1968 (Crustacea: Decapoda: Brachyura: Potamidae) from Zhuhai, Guangdong Province, China*. Zootaxa 3588(1):* 55–63. |
| Zhu et al. 2010 | Zhu C, Naruse T, Zhou XM (2010). Two New Species of Freshwater Crabs of the genus *Sinolapotamon* Tai & Sung, 1975 (Decapoda, Brachyura, Potamidae) from Guangxi Zhuang autonomous region, China. Crustaceana 83(2): 245–256. |
| Yeo et al. 2007 | Yeo DCJ, Naruse T (2007). A Revision of the Freshwater Crab Genus *Hainanpotamon* Dai, 1995 (Crustacea: Decapoda: Brachyura: Potamidae: Potamiscinae), with a Redescription of *Potamon (Potamon) orientale* (Parisi, 1916) and descriptions of three New Species. Zoological Science 24(11): 1143–1158. |
| Dai 1999 | Dai AY (1999). *Fauna Sinica. Arthropoda: Crustacea: Malacostraca: Decapoda: Parathelphusidae, Potamidae*. Science Press, Beijing (In Chinese with English abstract). |
| Dai 1995 | Dai AY (1995). On a new genus and two new species of freshwater crabs from Hainan Island, China (Crustacea: Decapoda: Brachyura: Potamidae). *Acta* Zootaxonomica Sinica 20: 391–397. |
| Ng and Dudgeon 1992 | Ng PKL, Dudgeon D (1992). The Potmidae and Parathelphusidae (Crustacea: Decapoda: Brachyura) of Hong Kong. Invertebrate Systematics 6(3): 741–768. |

**Table S2.** Ecological factors in this study.

| Variable types | Ecological factors | Abbreviation | References |
| --- | --- | --- | --- |
| Bioclimate | Annual Mean Temperature | Bio1 | Stephen and Hijmans 2017 |
| Bioclimate | Mean Diurnal Range (Mean of monthly (max temp - min temp)) | Bio2 | Stephen and Hijmans 2017 |
| Bioclimate | Isothermality (Bio2/Bio7) (×100) | Bio3 | Stephen and Hijmans 2017 |
| Bioclimate | Temperature Seasonality (standard deviation ×100) | Bio4 | Stephen and Hijmans 2017 |
| Bioclimate | Max Temperature of Warmest Month | Bio5 | Stephen and Hijmans 2017 |
| Bioclimate | Min Temperature of Coldest Month | Bio6 | Stephen and Hijmans 2017 |
| Bioclimate | Temperature Annual Range (Bio5-Bio6) | Bio7 | Stephen and Hijmans 2017 |
| Bioclimate | Mean Temperature of Wettest Quarter | Bio8 | Stephen and Hijmans 2017 |
| Bioclimate | Mean Temperature of Driest Quarter | Bio9 | Stephen and Hijmans 2017 |
| Bioclimate | Mean Temperature of Warmest Quarter | Bio10 | Stephen and Hijmans 2017 |
| Bioclimate | Mean Temperature of Coldest Quarter | Bio11 | Stephen and Hijmans 2017 |
| Bioclimate | Annual Precipitation | Bio12 | Stephen and Hijmans 2017 |
| Bioclimate | Precipitation of Wettest Month | Bio13 | Stephen and Hijmans 2017 |
| Bioclimate | Precipitation of Driest Month | Bio14 | Stephen and Hijmans 2017 |
| Bioclimate | Precipitation Seasonality (Coefficient of Variation) | Bio15 | Stephen and Hijmans 2017 |
| Bioclimate | Precipitation of Wettest Quarter | Bio16 | Stephen and Hijmans 2017 |
| Bioclimate | Precipitation of Driest Quarter | Bio17 | Stephen and Hijmans 2017 |
| Bioclimate | Precipitation of Warmest Quarter | Bio18 | Stephen and Hijmans 2017 |
| Bioclimate | Precipitation of Coldest Quarter | Bio19 | Stephen and Hijmans 2017 |
| Landscape | Altitude | ALT | This study established |
| Landscape | Slope | SLO | This study established |
| Landscape | Aspect | ASP | This study established |
| Human activities | Crop | CR | Wang et al. 2015 |
| Human activities | Footprint | FP | Sanderson et al. 2002 |
| Human activities | Urban | UR | Wang et al. 2015 |
| Land cover | Forest | FR | Wang et al. 2015 |
| Land cover | Grassland | GR | Wang et al. 2015 |
| Land cover | Shrub | SH | Wang et al. 2015 |
| Land cover | Others | OT | Wang et al. 2015 |

**Table S3.** Correlation matrix of ecological variables (r < 0.75).

| Variables group | Abbreviation | ALT | ASP | CR | FR | Bio1 | Bio2 | Bio4 | Bio12 | Bio14 |
| --- | --- | --- | --- | --- | --- | --- | --- | --- | --- | --- |
| Terrain | ALT | 1.00 | -0.04 | 0.01 | -0.21 | -0.19 | 0.61 | -0.44 | -0.35 | -0.34 |
|  | ASP | -0.04 | 1.00 | -0.23 | -0.17 | 0.00 | 0.09 | -0.17 | 0.18 | 0.04 |
| Land cover | CR | -0.03 | 0.26 | 1.00 | 0.11 | 0.03 | 0.18 | 0.11 | -0.21 | -0.15 |
|  | FR | 0.05 | 0.08 | 0.03 | 1.00 | 0.04 | -0.01 | 0.05 | 0.11 | -0.02 |
| Temperature | Bio1 | -0.19 | 0.00 | 0.26 | 0.38 | 1.00 | -0.42 | -0.13 | 0.08 | 0.13 |
|  | Bio2 | 0.61 | 0.09 | 0.19 | 0.03 | -0.42 | 1.00 | -0.04 | -0.42 | -0.44 |
|  | Bio4 | -0.44 | -0.17 | 0.31 | 0.25 | -0.13 | -0.04 | 1.00 | -0.12 | 0.13 |
| Precipitation | Bio12 | -0.35 | 0.18 | -0.11 | 0.06 | 0.08 | -0.42 | -0.12 | 1.00 | 0.68 |
|  | Bio14 | -0.34 | 0.04 | 0.13 | 0.07 | 0.13 | -0.44 | 0.13 | 0.68 | 1.00 |

ALT, altitude, ASP, aspect, CR, crop, FR, forest, Bio1, annual mean temperature, Bio2, mean diurnal range (mean of monthly (max temp - min temp)), Bio4, temperature seasonality (standard deviation ×100), Bio12, annual precipitation, and Bio14, precipitation of driest month.

**Table S4.** The variance inflation factor of nine selected variables (VIF < 5).

| Variables group | Abbreviation | Variance inflation factor |
| --- | --- | --- |
| Terrain | ALT | 3.2176 |
|  | ASP | 2.8451 |
| Land cover | CR | 3.1094 |
|  | FR | 2.7958 |
| Temperature | Bio1 | 3.2564 |
|  | Bio2 | 4.1123 |
|  | Bio4 | 2.0415 |
| Precipitation | Bio12 | 2.4123 |
|  | Bio14 | 2.0015 |

ALT, altitude, ASP, aspect, CR, crop, FR, forest, Bio1, annual mean temperature, Bio2, mean diurnal range (mean of monthly (max temp - min temp)), Bio4, temperature seasonality (standard deviation ×100), Bio12, annual precipitation, and Bio14, precipitation of driest month.

**Table S5.** A checklist of freshwater crabs in the STZC. Extent of occurrence (EOO) and area of occupancy (AOO); Nine categories: Not Evaluated (NE), Extinct (EX), Extinct in the Wild (EW), Critically Endangered (CR), Endangered (EN), Vulnerable (VU), Near Threatened (NT), Least Concern (LC), and Data Deficient (DD); The arrows represent rising (**↑**) and falling (**↓**); endemic genus or species in China (*).

| **Family** | **Genus** | | **Species (Subspecies)** | | **Historical results** | **Number of sites** | **EOO**  **(km^2^)** | **AOO**  **(km^2^)** | **Assessment status** | **Status change**  **[↑, ↓ or new]** | **Assessment criteria** |
| --- | --- | --- | --- | --- | --- | --- | --- | --- | --- | --- | --- |
| Gecarcinucidae  (2 genera) | 1 | *Mekhongthelphusa* | 1 | *M. menglongensis** | NE | 1 | 4155 | 30 | NT |  |  |
|  | 2 | *Somanniathelphusa* | 2 | *S. amoyensis** | DD | 2 | 5518 | 60 | NT | new |  |
|  |  |  | 3 | *S. araeochela** | DD | 3 | 399 | 90 | NT | new |  |
|  |  |  | 4 | *S. bawangensis** | DD | 12 | 6368 | 600 | LC | new |  |
|  |  |  | 5 | *S. brevipodum** | LC | 9 | 48000 | 450 | LC |  |  |
|  |  |  | 6 | *S. chongi** | LC | 15 | 27839 | 750 | LC |  |  |
|  |  |  | 7 | *S. falx** | DD | 5 | 4404 | 250 | LC | new |  |
|  |  |  | 8 | *S. hainanensis** | DD | 10 | 5612 | 500 | LC | new |  |
|  |  |  | 9 | *S. huaanensis** | DD | 3 | 1203 | 90 | NT | new |  |
|  |  |  | 10 | *S. longicaudus** | DD | 2 | 1501 | 100 | NT | new |  |
|  |  |  | 11 | *S. megachela** | DD | 2 | 399 | 60 | NT | new |  |
|  |  |  | 12 | *S. nanningensis** | DD | 1 | 1501 | 30 | DD |  |  |
|  |  |  | 13 | *S. qiongshanensis** | DD | 10 | 5490 | 500 | LC | new |  |
|  |  |  | 14 | *S. sinensis** | DD | 3 | 5933 | 90 | NT | new |  |
|  |  |  | 15 | *S. taiwanensis** | NE | 5 | 259 | 150 | NT |  |  |
|  |  |  | 16 | *S. tongzhaensis** | DD | 15 | 9840 | 450 | LC | new |  |
|  |  |  | 17 | *S. yuilinensis** | DD | 19 | 80034 | 450 | LC | new |  |
|  |  |  | 18 | *S. zanklon** | LC | 13 | 1850 | 390 | LC |  |  |
|  |  |  | 19 | *S. zhangpuensis** | DD | 2 | 13328 | 60 | DD |  |  |
|  |  |  | 20 | *S. zhapoensis** | DD | 1 | 1364 | 30 | DD |  |  |
| Potamidae  (43 genera) | 3 | *Aiyunamon* | 21 | *A. daiae** | LC | 2 | 500 | 60 | VU | ↑ | B1ab(i,ii,iii,iv)+2ab(i,ii,iii,iv) |
|  |  |  | 22 | *A. fatum** | NE | 4 | 776 | 120 | LC |  |  |
|  |  |  | 23 | *A. lushuiense** | LC | 8 | 308 | 240 | LC |  |  |
|  |  |  | 24 | *A. tengchongense** | LC | 4 | 2816 | 120 | LC |  |  |
|  | 4 | *Aparapotamon** | 25 | *A. grahami* | LC | 50 | 511673 | 1500 | LC |  |  |
|  |  |  | 26 | *A. similium* | VU | 3 | 200 | 900 | VU |  |  |
|  | 5 | *Apotamonautes** | 27 | *A. hainanensis banshuiensis* | NE | 2 | 171 | 60 | NT |  |  |
|  |  |  | 28 | *A. hainanensis bawanglingensis* | NE | 44 | 6513 | 1320 | LC |  |  |
|  |  |  | 29 | *A. hainanensis hainanensis* | NE | 5 | 4795 | 150 | NT |  |  |
|  |  |  | 30 | *A. hainanensis nanlinensis* | NE | 5 | 824 | 150 | NT |  |  |
|  | 6 | *Barbamon** | 31 | *B. zhoui* | NE | 1 | 171 | 10 | EN |  | B1ab(i,ii,iii,iv)+2ab(i,ii,iii,iv) |
|  | 7 | *Bottapotamon** | 32 | *B. nanan* | NE | 11 | 38255 | 220 | LC |  |  |
|  | 8 | *Calcipotamon** | 33 | *C. puglabrum* | EN | 1 | 164 | 10 | EN |  | B1ab(i,ii,iii,iv)+2ab(i,ii,iii,iv) |
|  | 9 | *Candidiopotamon* | 34 | *C. rathbuni** | NE | 39 | 5714 | 1170 | LC |  |  |
|  |  |  | 35 | *C. penglai** | NE | 16 | 2857 | 480 | LC |  |  |
|  | 10 | *Cantopotamon** | 36 | *C. hengqinense* | EN | 3 | 1504 | 90 | VU | ↓ | B1ab(i,ii,iii)+2ab(i,ii,iii) |
|  |  |  | 37 | *C. shangchuanense* | NE | 1 | 1732 | 30 | VU |  | B1ab(i,ii)+2ab(i,ii) |
|  |  |  | 38 | *C. yangxiense* | NE | 1 | 601 | 30 | EN |  | B1ab(i,ii,iii,iv)+2ab(i,ii,iii,iv) |
|  |  |  | 39 | *C. zhuhaiense* | NE | 1 | 1725 | 30 | VU |  | B1ab(i,ii)+2ab(i,ii) |
|  | 11 | *Chinapotamon** | 40 | *C. clarkei* | NE | 3 | 2645 | 90 | NT |  |  |
|  |  |  | 41 | *C. dashiwei* | NE | 3 | 200 | 90 | NT |  |  |
|  |  |  | 42 | *C. depressum* | LC | 15 | 129538 | 450 | LC |  |  |
|  |  |  | 43 | *C. glabrum* | DD | 5 | 13094 | 150 | LC | new |  |
|  |  |  | 44 | *C. longlinense* | DD | 4 | 4236 | 120 | NT | new |  |
|  |  |  | 45 | *C. maolanense* | NE | 1 | 200 | 30 | VU |  | D2 |
|  |  |  | 46 | *C. pusillum* | DD | 1 | 281 | 30 | EN | new | B1ab(i,ii)+2ab(i,ii) |
|  |  |  | 47 | *C. xingrenense* | DD | 5 | 16438 | 150 | NT | new |  |
|  | 12 | *Daipotamon** | 48 | *D. minos* | LC | 2 | 4 | \ | EN | ↑ | B1ab(i,ii,iii,iv)+2ab(i,ii,iii,vi) |
|  | 13 | *Eurusamon** | 49 | *E. guangdongense* | DD | 6 | 32780 | 180 | NT | new |  |
|  | 14 | *Geothelphusa* | 50 | *G. albogilva** | LC | 19 | 1029 | 570 | LC |  |  |
|  |  |  | 51 | *G. ancylophallus** | NE | 7 | 678 | 210 | LC |  |  |
|  |  |  | 52 | *G. bicolor** | LC | 12 | 2216 | 360 | LC |  |  |
|  |  |  | 53 | *G. boreas** | NE | 1 | 507 | 30 | DD |  |  |
|  |  |  | 54 | *G. caesia** | LC | 4 | 1289 | 120 | NT | ↑ |  |
|  |  |  | 55 | *G. candidiensis** | LC | 5 | 1563 | 150 | LC |  |  |
|  |  |  | 56 | *G. chiui** | DD | 2 | 316 | 60 | VU | new | B1ab(i,ii)+2ab(i,ii) |
|  |  |  | 57 | *G. cilan** | NE | 1 | 192 | 30 | DD |  |  |
|  |  |  | 58 | *G. cinerea** | LC | 10 | 1946 | 300 | LC |  |  |
|  |  |  | 59 | *G. dolichopodes** | LC | 7 | 927 | 210 | LC |  |  |
|  |  |  | 60 | *G. eucrinodonta** | NE | 17 | 4382 | 510 | LC |  |  |
|  |  |  | 61 | *G. eurysoma** | DD | 6 | 4267 | 180 | LC | new |  |
|  |  |  | 62 | *G. ferruginea** | DD | 7 | 142 | 210 | LC | new |  |
|  |  |  | 63 | *G. gracilipes** | DD | 4 | 621 | 120 | NT | new |  |
|  |  |  | 64 | *G. haituan** | NE | 2 | 136 | 60 | VU |  | B1ab(i,ii)+2ab(i,ii) |
|  |  |  | 65 | *G. hirsuta** | NE | 4 | 1220 | 120 | NT |  |  |
|  |  |  | 66 | *G. holthuisi** | NE | 1 | 204 | 30 | DD |  |  |
|  |  |  | 67 | *G. ilan** | NE | 9 | 3016 | 270 | LC |  |  |
|  |  |  | 68 | *G. lanyu** | CR | 5 | 433 | 150 | VU | ↓ |  |
|  |  |  | 69 | *G. leeae** | DD | 1 | 396 | 30 | DD |  |  |
|  |  |  | 70 | *G. lili** | NE | 4 | 1047 | 120 | LC |  |  |
|  |  |  | 71 | *G. makatao** | NE | 4 | 285 | 120 | LC |  |  |
|  |  |  | 72 | *G. miyazakii** | NT | 10 | 674 | 300 | LC | ↓ |  |
|  |  |  | 73 | *G. monticola** | NE | 6 | 140 | 180 | LC |  |  |
|  |  |  | 74 | *G. nanao** | NE | 4 | 410 | 120 | LC |  |  |
|  |  |  | 75 | *G. nanhsi** | NE | 5 | 1151 | 150 | LC |  |  |
|  |  |  | 76 | *G. olea** | LC | 31 | 5032 | 930 | LC |  |  |
|  |  |  | 77 | *G. pingtung** | VU | 4 | 488 | 120 | VU |  |  |
|  |  |  | 78 | *G. shernshan** | NE | 3 | 420 | 90 | NT |  |  |
|  |  |  | 79 | *G. shokitai* | LC | 1 | 248 | 30 | VU | ↑ | B1ab(i,ii)+2ab(i,ii) |
|  |  |  | 80 | *G. siasiat** | NE | 2 | 871 | 60 | VU |  |  |
|  |  |  | 81 | *G. takuan** | NE | 1 | 396 | 30 | DD |  |  |
|  |  |  | 82 | *G. tali** | DD | 4 | 1079 | 120 | LC | new |  |
|  |  |  | 83 | *G. taroko** | NE | 3 | 886 | 90 | VU |  |  |
|  |  |  | 84 | *G. tawu** | DD | 11 | 2596 | 330 | LC | new |  |
|  |  |  | 85 | *G. tsayae** | NE | 5 | 2005 | 150 | LC |  |  |
|  |  |  | 86 | *G. wangi** | VU | 1 | 118 | 30 | VU |  |  |
|  |  |  | 87 | *G. wutai** | VU | 2 | 420 | 60 | VU |  |  |
|  |  |  | 88 | *G. yangmingshan** | NE | 2 | 383 | 60 | VU |  |  |
|  | 15 | *Hainanpotamon* | 89 | *H. daiae** | DD | 16 | 11596 | 480 | LC | new |  |
|  |  |  | 90 | *H. fuchengense** | DD | 4 | 904 | 120 | NT | new |  |
|  |  |  | 91 | *H. helense** | DD | 9 | 3028 | 270 | LC | new |  |
|  |  |  | 92 | *H. orientale** | EN | 8 | 5160 | 240 | NT | ↓ |  |
|  | 16 | *Heterochelamon** | 93 | *H. castanea* | NE | 2 | 138 | 60 | VU |  |  |
|  |  |  | 94 | *H. guangxiense* | DD | 1 | 489 | 30 | DD |  |  |
|  |  |  | 95 | *H. huidongense* | NE | 3 | 3028 | 90 | NT |  |  |
|  |  |  | 96 | *H. purpureomanuale* | DD | 1 | 32 | 30 | VU | new | D2 |
|  |  |  | 97 | *H. tessellatum* | NE | 4 | 692 | 120 | NT |  |  |
|  | 17 | *Huananpotamon** | 98 | *H. angulatum* | DD | 7 | 6533 | 140 | LC | new |  |
|  |  |  | 99 | *H. changzhium* | NE | 2 | 3424 | 40 | NT |  |  |
|  |  |  | 100 | *H. planopodum* | DD | 10 | 14135 | 200 | LC | new |  |
|  |  |  | 101 | *H. zhangzhouense* | DD | 6 | 588 | 120 | LC | new |  |
|  | 18 | *Indochinamon* | 102 | *I. ahkense** | NE | 2 | 201 | 60 | NT |  |  |
|  |  |  | 103 | *I. andersonianum* | DD | 2 | 1322 | 60 | NT | new |  |
|  |  |  | 104 | *I. boshanense** | LC | 4 | 14524 | 120 | LC |  |  |
|  |  |  | 105 | *I. changpoense** | DD | 3 | 3781 | 90 | NT | new |  |
|  |  |  | 106 | *I. chinghungense** | DD | 28 | 7218 | 540 | LC | new |  |
|  |  |  | 107 | *I. daweishanense** | LC | 7 | 1245 | 210 | LC |  |  |
|  |  |  | 108 | *I. edwardsi* | LC | 9 | 8786 | 280 | LC |  |  |
|  |  |  | 109 | *I. flexum** | DD | 4 | 1217 | 120 | LC | new |  |
|  |  |  | 110 | *I. gengmaense** | DD | 11 | 2613 | 330 | LC | new |  |
|  |  |  | 111 | *I. hispidum* | DD | 5 | 10967 | 150 | LC | new |  |
|  |  |  | 112 | *I. jianchuanense** | LC | 5 | 3160 | 150 | LC |  |  |
|  |  |  | 113 | *I. jinpingense* | DD | 9 | 14726 | 270 | LC | new |  |
|  |  |  | 114 | *I. khinpyae* | LC | 2 | 209 | 60 | LC |  |  |
|  |  |  | 115 | *I. lui** | LC | 18 | 20183 | 540 | LC |  |  |
|  |  |  | 116 | *I. malipoense** | NE | 6 | 2637 | 180 | LC |  |  |
|  |  |  | 117 | *I. menglaense** | NE | 3 | 7418 | 90 | LC |  |  |
|  |  |  | 118 | *I. parpidum** | NE | 2 | 1057 | 60 | NT |  |  |
|  |  |  | 119 | *I. tannanti* | DD | 10 | 24196 | 300 | LC | new |  |
|  |  |  | 120 | *I. tujiense** | LC | 4 | 8248 | 120 | LC |  |  |
|  |  |  | 121 | *I. xinpingense** | LC | 6 | 19869 | 180 | LC |  |  |
|  |  |  | 122 | *I. frontatum** | NE | 1 | 1214 | 30 | NT |  |  |
|  | 19 | *Lacunipotamon** | 123 | *L. albusorbitum* | DD | 1 | 88 | 20 | VU | new | D2 |
|  |  |  | 124 | *L. cymatile* | NE | 1 | 183 | 20 | VU |  | B1ab(i,ii,iii)+2ab(i,ii,iii) |
|  |  |  | 125 | *L. yuanshi* | NE | 4 | 247 | 80 | NT |  |  |
|  | 20 | *Luteomon** | 126 | *L. spinapodum* | NE | 1 | 558 | 20 | VU |  | B1ab(i,ii,iii)+2ab(i,ii,iii) |
|  | 21 | *Mediapotamon** | 127 | *M. angustipedum* | DD | 1 | 946 | 30 | NT | new |  |
|  | 22 | *Megapleonum** | 128 | *M. ehuangzhang* | NE | 1 | 602 | 30 | NT |  |  |
|  |  |  | 129 | *M. shenzhen* | NE | 1 | 1950 | 30 | NT |  |  |
|  | 23 | *Minpotamon** | 130 | *M. auritum* | NE | 2 | 1091 | 40 | NT |  |  |
|  |  |  | 131 | *M. kityang* | NE | 1 | 1632 | 20 | NT |  |  |
|  |  |  | 132 | *M. nasicum* | NE | 1 | 4809 | 20 | NT |  |  |
|  | 24 | *Minutomon** | 133 | *M. shanweiense* | NE | 3 | 1514 | 60 | NT |  |  |
|  | 25 | *Nanhaipotamon** | 134 | *N. aculatum* | DD | 2 | 3782 | 60 | NT | new |  |
|  |  |  | 135 | *N. aureomarginatum* | NE | 3 | 3231 | 90 | LC |  |  |
|  |  |  | 136 | *N. formosanum* | VU | 19 | 6490 | 570 | LC | ↓ |  |
|  |  |  | 137 | *N. guangdongense* | LC | 1 | 5326 | 30 | DD |  |  |
|  |  |  | 138 | *N. hongkongense* | LC | 3 | 4666 | 90 | NT | ↑ |  |
|  |  |  | 139 | *N. huaanense* | DD | 2 | 588 | 60 | NT | new |  |
|  |  |  | 140 | *N. incendium* | NE | 2 | 1681 | 60 | NT |  |  |
|  |  |  | 141 | *N. longhaiense* | NE | 3 | 497 | 90 | LC |  |  |
|  |  |  | 142 | *N. macau* | NT | 1 | 1504 | 30 | EN | ↑ |  |
|  |  |  | 143 | *N. nanriense* | DD | 8 | 4635 | 240 | NT | new |  |
|  |  |  | 144 | *N. pingtanense* | NE | 2 | 713 | 60 | NT |  |  |
|  |  |  | 145 | *N. pingyuanense* | DD | 3 | 1120 | 90 | LC | new |  |
|  |  |  | 146 | *N. yongchuense* | DD | 3 | 4491 | 90 | LC | new |  |
|  |  |  | 147 | *N. zhuhaiense* | NE | 2 | 1505 | 60 | NT |  |  |
|  | 26 | *Neotiwaripotamon** | 148 | *N. jianfengense* | DD | 15 | 4635 | 450 | LC | new |  |
|  |  |  | 149 | *N. whiteheadi* | DD | 5 | 1810 | 150 | NT | new |  |
|  | 27 | *Parapotamon** | 150 | *P. spinescens* | VU | 4 | 30918 | 160 | LC | ↓ |  |
|  | 28 | *Pararanguna** | 151 | *P. hemicyclia* | EN | 2 | 940 | 40 | EN |  | B1ab(i,ii,iii,iv)+2ab(i,ii,iii) |
|  |  |  | 152 | *P. semilunatum* | LC | 2 | 3000 | 40 | VU | ↑ | D2 |
|  | 29 | *Paratelphusula* | 153 | *P. burmensis* | DD | 1 | 359 | 30 | LC | new |  |
|  | 30 | *Parvuspotamon** | 154 | *P. yuxiense* | VU | 2 | 539 | 60 | VU |  | B1ab(i,ii)+2ab(i,ii,iii) |
|  | 31 | *Phasmon** | 155 | *P. typhlops* | NE | 1 | 1190 | 30 | EN |  | B1ab(i,ii,iii,iv)+2ab(i,ii,iii) |
|  | 32 | *Potamiscus* | 156 | *P. cangyuanensis** | DD | 1 | 766 | 30 | VU | new | B1ab(i,ii)+2ab(i,ii,iii) |
|  |  |  | 157 | *P. montosus** | DD | 3 | 1593 | 90 | NT | new |  |
|  |  |  | 158 | *P. yiwuensis** | DD | 3 | 1264 | 90 | NT | new |  |
|  | 33 | *Pusillamon** | 159 | *P. baishuiense* | DD | 2 | 260 | 20 | VU | new | D2 |
|  |  |  | 160 | *P. huaningense* | DD | 2 | 230 | 20 | VU | new | D2 |
|  |  |  | 161 | *P. panxiense* | DD | 1 | 27 | 10 | VU | new | D2 |
|  |  |  | 162 | *P. tonghaiense* | DD | 3 | 875 | 30 | NT | new |  |
|  |  |  | 163 | *P. xinpingense* | DD | 2 | 235 | 20 | VU | new | B1ab(i,ii,iii)+2ab(i,ii,iii) |
|  |  |  | 164 | *P. yuxiense* | DD | 2 | 2415 | 20 | NT | new |  |
|  | 34 | *Qianguimon** | 165 | *Q. aflagellum* | DD | 4 | 240 | 80 | LC | new |  |
|  |  |  | 166 | *Q. rongxianense* | NE | 1 | 1099 | 20 | DD | ↓ |  |
|  |  |  | 167 | *Q. splendidum* | NE | 1 | 138 | 20 | DD | ↓ |  |
|  |  |  | 168 | *Q. yuzhouense* | NE | 1 | 877 | 20 | DD | ↓ |  |
|  | 35 | *Semicirculara** | 169 | *S. lincangensis* | NE | 4 | 2926 | 120 | NT |  |  |
|  | 36 | *Sinolapotamon** | 170 | *S. auriculatum* | NE | 3 | 1246 | 20 | NT |  |  |
|  |  |  | 171 | *S. anacoluthon* | VU | 1 | 50 | 20 | VU |  | B1ab(i,ii,iii)+2ab(i,ii,iii) |
|  |  |  | 172 | *S. cirratum* | NE | 2 | 1117 | 60 | NT |  |  |
|  |  |  | 173 | *S. palmatum* | DD | 3 | 1074 | 90 | NT | new |  |
|  |  |  | 174 | *S. patellifer* | LC | 5 | 11286 | 150 | LC |  |  |
|  | 37 | *Sinopotamon** | 175 | *S. anyuanense* | LC | 15 | 20683 | 450 | LC |  |  |
|  |  |  | 176 | *S. exiguum* | LC | 23 | 37 | 690 | LC |  |  |
|  |  |  | 177 | *S. fukienense* | LC | 60 | 2582 | 1800 | LC |  |  |
|  |  |  | 178 | *S. jianglense* | LC | 12 | 9094 | 360 | LC |  |  |
|  |  |  | 179 | *S. longlinense* | DD | 3 | 179 | 90 | LC | new |  |
|  |  |  | 180 | *S. pinheensis* | NE | 4 | 9439 | 120 | LC |  |  |
|  |  |  | 181 | *S. rongshuiense* | LC | 9 | 14744 | 270 | LC |  |  |
|  |  |  | 182 | *S. zhangzhouense* | NE | 2 | 1082 | 60 | NT |  |  |
|  | 38 | *Songpotamon** | 183 | *S. funingense* | NE | 2 | 532 | 60 | NT |  |  |
|  |  |  | 184 | *S. malipoense* | NE | 1 | 476 | 30 | VU |  | B1ab(i,ii)+2ab(i,ii,iii) |
|  |  |  | 185 | *S. dixuense* | NT | 8 | 1904 | 240 | LC | ↑ |  |
|  | 39 | *Tenuipotamon** | 186 | *T. purpura* | DD | 2 | 1316 | 40 | EN | new | B1ab(i,ii,iii,iv)+2ab(i,ii,iii,iv) |
|  | 40 | *Teoswamon** | 187 | *T. scolasticum* | NE | 3 | 1278 | 60 | VU |  | B1ab(i,ii,iii)+2ab(i,ii,iii) |
|  | 41 | *Teretamon* | 188 | *T. husicum** | NE | 1 | 1213 | 20 | EN |  | B1ab(i,ii,iii,iv)+2ab(i,ii,iii,iv) |
|  | 42 | *Tiwaripotamon* | 189 | *T. pingguoense** | DD | 1 | 1543 | 30 | NT | new |  |
|  |  |  | 190 | *T. pluviosum* | DD | 2 | 1477 | 60 | NT | new |  |
|  | 43 | *Tortomon* | 191 | *T. gejiu** | NE | 3 | 495 | 90 | NT |  |  |
|  |  |  | 192 | *T. puer** | NE | 1 | 437 | 30 | EN |  | B1ab(i,ii,iii,iv)+2ab(iii,iv) |
|  | 44 | *Trichopotamon** | 193 | *T. daliense* | EN | 3 | 12 | 90 | VU |  | B1ab(i,ii)+2ab(i,ii,iii) |
|  | 45 | *Yarepotamon** | 194 | *Y. breviflagellum* | DD | 6 | 13281 | 180 | LC | new |  |
|  |  |  | 195 | *Y. gracillipa* | DD | 3 | 102 | 90 | LC | new |  |

**Table S6.** Summary of species in each diversity hotspot (* and ** indicate Vulnerable (VU) and Endangered (EN) status, respectively). Hotspot 1, Yongde Daisetsuzan and the surrounding area (YDSA); Hotspot 2, Wuliang Mountains and Ailao Mountains region (WAM); Hotspot 3, Dai Autonomous Prefecture of Xishuangbanna (DAPX); Hotspot 4, southern Ailao Mountains and lower reaches of Yuanjiang River (SAMY); Hotspot 5, County-Maguan and County-Malipo County in Wenshan Prefecture(MMWP); Hotspot 6, Wuyishan Mountains (WYS); Hotspot 7, karst areas in Guangxi and Guizhou (KAGG); Hotspot 8, Pearl River Delta, Hong Kong, and Macau (PHM); Hotspot 9, southwestern part of Hainan Island (HI); Hotspot 10 and 11, northwestern and southern terminus of Central Mountains in Taiwan (NCMT and SCMT, respectively)

| Hotspots | *genera* | *Species* |
| --- | --- | --- |
| 1 (YDSA) | *Aiyunamon* | *A. fatum* |
|  | *Indochinamon* | *I. hispidum; I. lui; I. gengmaense; I. edwardsii* |
|  | *Parapotamon* | *P. spinescens* |
|  | *Pararanguna* | *P. hemicyclia *; P. semilunata* |
|  | *Potamiscus* | *P. cangyuanensis ** |
|  | *Semicirculara* | *S. lincangensis* |
|  | *Somanniathelphusa* | *S. chongi* |
| 2 (WAM) | *Aparapotamon* | *A. similium ** |
|  | *Indochinamon* | *I. boshanense; I. xinpingense; I. lui; I. daweishanense; I. tujiense; I. parpidum* |
|  | *Parapotamon* | *P. spinescens* |
|  | *Parvuspotamon* | *P. yuxiense* |
|  | *Pusillamon* | *P. yuxiense; P. tonghaiense; P. panxiense *; P. xingpingense *; P. huaningense *; P. baishuiense ** |
|  | *Somanniathelphusa* | *S. chongi; S. brevipodum* |
| 3 (DAPX) | *Indochinamon* | *I. chinghungense; I. hispidum; I. daweishanense; I. menglaense; I. lui* |
|  | *Tenuipotamon* | *T. purpura *** |
|  | *Somanniathelphusa* | *S. chongi; S. brevipodum* |
|  | *Potamiscus* | *P. montosus; P. yiwuensis* |
|  | *Mekhongthelphusa* | *M. menglongensis* |
|  | *Tortomon* | *T. puer *** |
| 4 (SAMY) | *Indochinamon* | *I. parpidum; I. chinghungense; I. tannanti; I. jinpingense; I. changpoense; I. daweishanense* |
|  | *Parapotamon* | *P. spinescens* |
|  | *Somanniathelphusa* | *S. brevipodum* |
|  | *Tortomon* | *T. gejiu* |
| 5 (MMWP) | *Indochinamon* | *I. tannanti; I. malipoense* |
|  | *Barbamon* | *B. zhoui *** |
|  | *Lacunipotamon* | *L. yuanshi* |
|  | *Chinapotamon* | *C. depressum* |
|  | *Somanniathelphusa* | 1. *brevipodum* |
| 6 (WYS) | *Bottpotamon* | *B. nanan* |
|  | *Huananpotamon* | *H. planopodum* |
|  | *Minpotamon* | *M. auritum; M. nasicum* |
|  | *Sinopotamon* | *S. jianglense; S. zhangzhouense; S. pinheensis* |
|  | *Nanhaipotamon* | *N. yongchuense; N. huaanense; N. longhaiense* |
| 7 (KAGG) | *Chinapotamon* | 1. *glabrum; C. charkei; C. depressum; C. dashiwei; C. anlongense; C. pusillum *** |
|  | *Heterochelamon* | *H. guangxiense; H. tessellatum; H. castanea ** |
|  | *Indochinamon* | *I. ahkense* |
|  | *Qianguimon* | *Q. aflagellum; Q. splendidum* |
|  | *Sinolapotamon* | *S. palmatum; S. auriculatum* |
|  | *Somanniathelphusa* | *S. longicaudus S. araeochela* |
| 8 (PHM) | *Cantopotamon* | *C. zhuhaiense; C. hengqinense ** |
|  | *Sinolapotamon* | *S. anacoluthon* |
|  | *Eurusamon* | *E. guangdongense* |
|  | *Nanhaipotamon* | *N. aculatum; N. hongkongense; N. zhuhaiense; N. macau *** |
|  | *Megapleonum* | *M. shenzhen* |
|  | *Somanniathelphusa* | *S. zanklon; S. anacoluthon ** |
| 9 (HI) | *Hainanpotamon* | *H. daiae; H. fuchengense; H. helense; H. orientale* |
|  | *Apotamonautes* | *A. hainanensis banshuiensis; A. hainanensis bawanglingensis; A. hainanensis hainanensis; A. hainanensis nanlinensis* |
|  | *Somanniathelphusa* | *S. banwangensis; S. hainanensis; S. tongzhaensis* |
|  | *Neotiwaripotamon* | *N. jianfengense; N. whiteheadi* |
|  | *Calcipotamon* | *C. puglabrum *** |
| 10 (SCMT) | *Geothelphusa* | *G. olea; G. takuan; G. hirsuta; G. eucrinodonta; G. caesia; G. siasiat ** |
|  | *Candidiopotamon* | *C. rathbuni* |
| 11 (NCMT) | *Geothelphusa* | *G. ancylophallus; G. albogilva; G. caesia; G. pingtung *; G. lili; G. wutai *; G. shernshan; G. olea; G. tsayae; G. tawu; G. lanyu *; G. ferruginea; G. holthuisi; G. makatao; G. nanhsi* |
|  | *Candidiopotamon* | *C. rathbuni,* |
|  | *Nanhaipotamon* | *N. formosanum* |
|  | *Somanniathelphusa* | *S. taiwanensis* |

**Table S7.** Descriptive statistics of MLR between richness and ecological variables.

|  | Estimate | Standard error | Statistic | *p* value |
| --- | --- | --- | --- | --- |
| ALT | 0.001 | 0.000 | 4.216 | *** |
| CR | 1.112 | 0.002 | -3.442 | *** |
| FR | 1.089 | 0.003 | 3.214 | *** |
| BIO1 | -0.010 | 0.001 | 2.234 | ** |
| BIO14 | 0.015 | 0.012 | -2.987 | ** |

ALT, altitude, CR, crop, FR, forest, Bio1, annual mean temperature, and Bio14, precipitation of driest month. Signif. codes: *** *p* < 0.001, ** *p* < 0.01; Residual standard error: 0.5393 on 330 degrees of freedom; Multiple R^2^ = 0.355, Adjusted R^2^ = 0.347; F-statistic = 45.36 on 330 degrees of freedom; *p* value < 0.001.

**Table S8.** Diagnostic Measure for multiple linear regression (MLR), and geographically weighted regression (GWR).

| Models | R^2^ | Adjusted R^2^ | AIC | AICc | RSS |
| --- | --- | --- | --- | --- | --- |
| GWR | 0.730 | 0.671 | 740.530 | 784.750 | 424.765 |
| MLR | 0.481 | 0.467 | 853.292 | 856.910 | 817.044 |

AIC, Akaike information criteria, AICc, Akaike Information Criterion corrected, and RSS, Residual Sum of Squares.

**Table S9.** Descriptive statistics of geographically weighted regression between richness and ecological variables.

|  | Minimum | 25% quartile | Median | 75% quartile | Maximum |
| --- | --- | --- | --- | --- | --- |
| ALT | -3.007E+01 | -1.615E+00 | 7.428E+00 | 1.384E+01 | 2.039E+01 |
| CR | -6.614E-03 | -1.304E-03 | -3.832E-04 | 3.384E-03 | 8.800E-03 |
| FR | -1.841E+00 | -9.642E-01 | -2.158E-01 | 6.663E-01 | 3.957E+00 |
| BIO1 | -1.170E-02 | -2.461E-03 | -8.913E-04 | 1.230E-03 | 9.900E-03 |
| BIO14 | -1.211E+00 | -2.122E-03 | -1.318E-01 | 5.221E-01 | 3.213E+01 |

ALT, altitude, CR, crop, FR, forest, Bio1, annual mean temperature, and Bio14, precipitation of driest month.


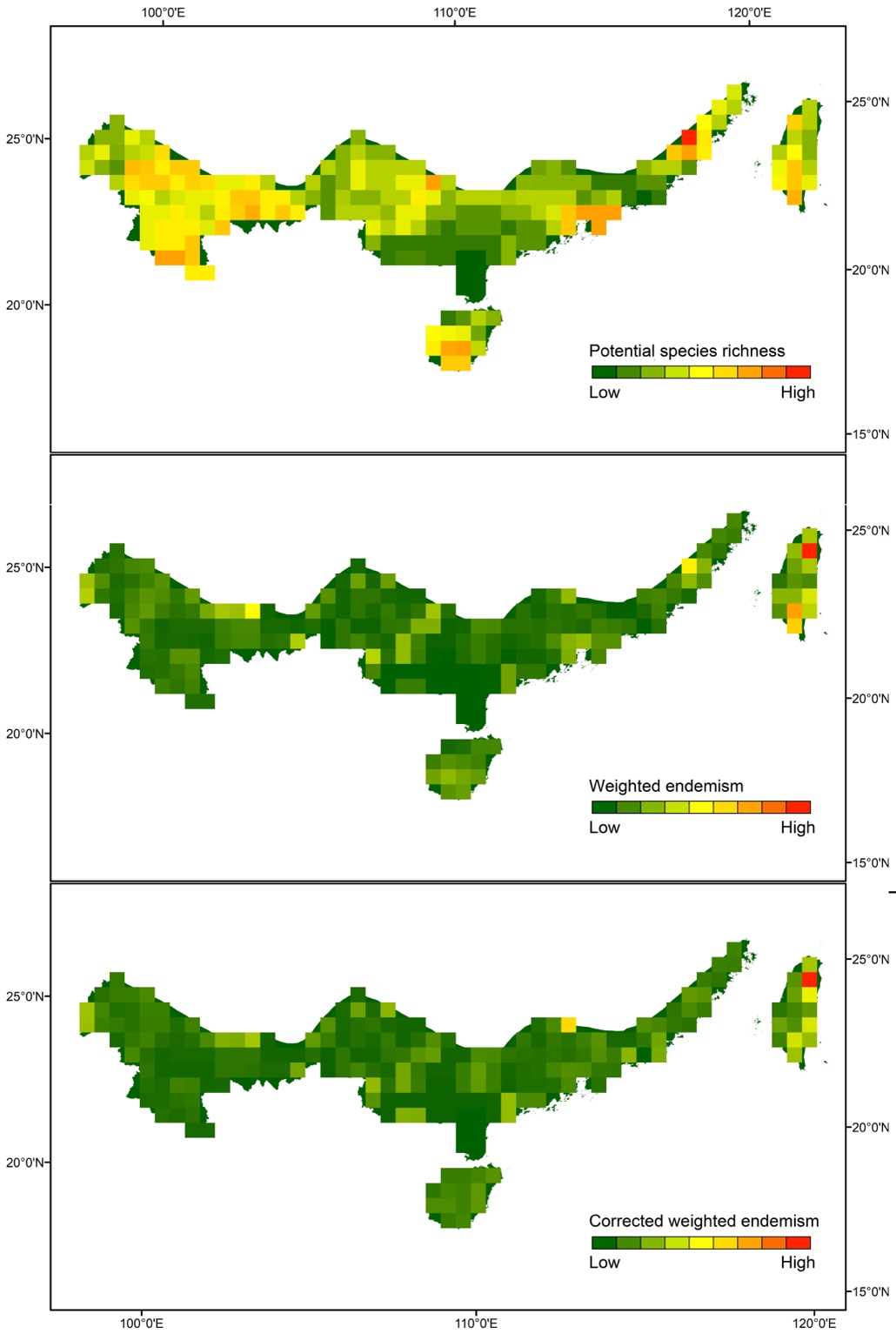
**Appendix B. Figure S1 and S2**

**FIGURE S1.** Potential richness pattern, weighted endemism, and corrected weighted endemism of freshwater crabs based on 0.5° grids (about 50 km).


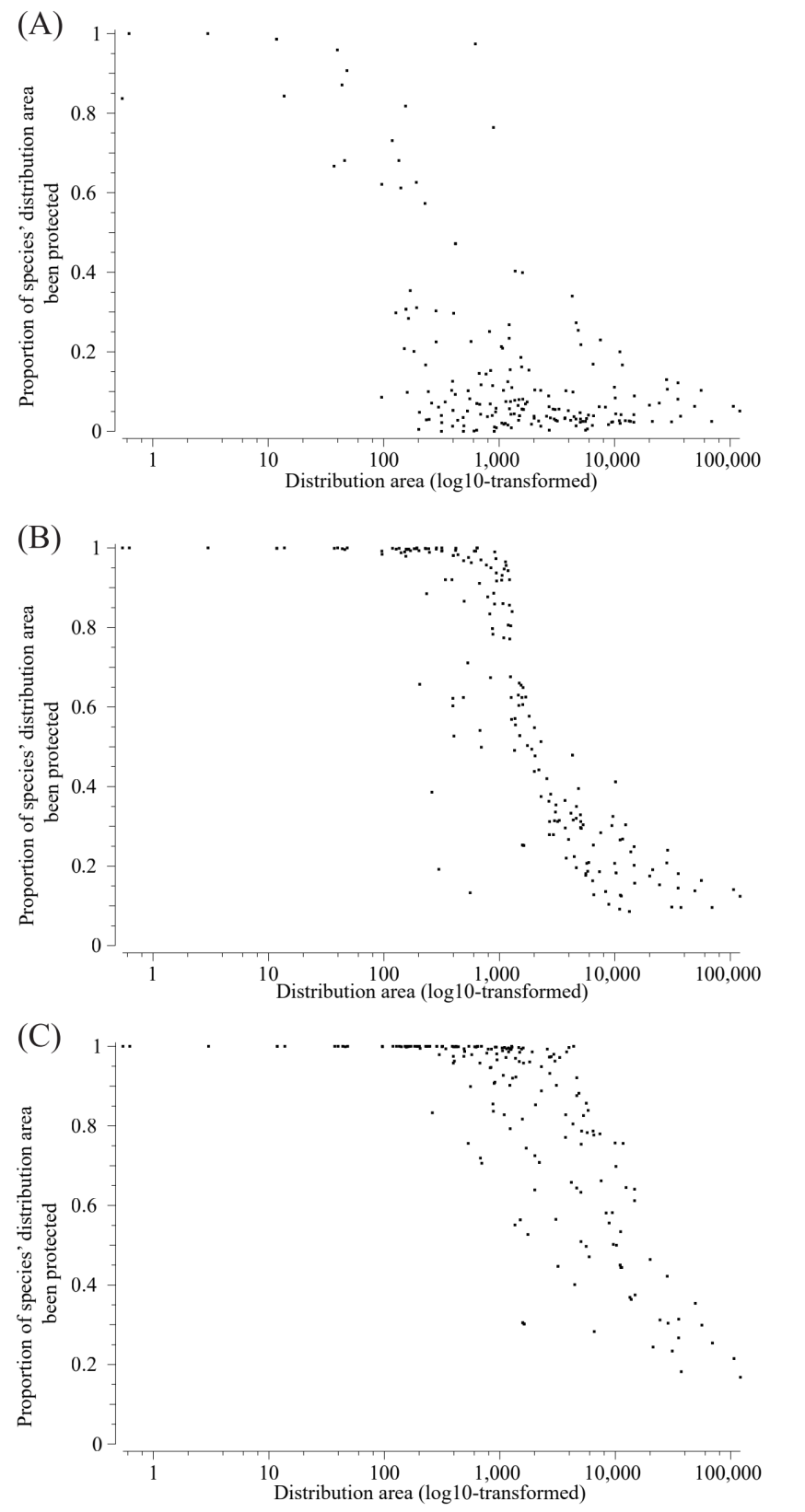


**FIGURE S2.** The proportion of each species distribution range protected by current NRs (A: 5.7% of the region) and potential conservation priority areas (B: 15% of the region; C: 30% of the region).

**Appendix C.**

**The details of freshwater crab species in hotspot 1-11.**

Hotspot 1 (YDSA) is home to seven genera and 11 species, including *Aiyunamon* (1 species), *Indochinamon* (4 species), *Parapotamon* (1 species), *Pararanguna* (2 species), *Potamiscus* (1 species), *Semicirculara* (1 species), and *Somanniathelphusa* (1 species). Species in this region are primarily found at high elevations, often residing beneath rocks in mountain streams. This area is also the most ecologically complex region in the Hengduan Mountains, where altitude variations and dry-hot river valleys contribute to its unique landscape, fostering species diversity and endemism. While altitude habitats may offer a refuge, it also carries the risk of isolating populations.

Hotspot 2 (WAM) is distributed along the two longitudinal mountain ranges, Wuliang Mountains and Ailao Mountains, exhibiting ribbon-like distributions. Six genera and 17 species are found in this area, including *Aparapotamon* (1 species), *Indochinamon* (6 species), *Parapotamon* (1 species), *Parvuspotamon* (1 species), *Pusillamon* (6 species), *Somanniathelphusa* (2 species). Species in this region exhibit significant morphological and habitat differences. For example, *Indochinamon*, *Parapotamon*, and *Somanniathelphusa* are larger and often found under rocks in mountain streams, while *Parvuspotamon*, *Aparapotamon*, and *Pusillamon*, which have smaller carapaces, typically burrow along streambanks. In addition, inhabiting in lowland streams and lakes, *Parapotamon* and *Somanniathelphusa* have developed sharp epibranchial teeth, which help protect them from predation.

Hotspot 3 (DAPX) encompasses most of the Dai Autonomous Prefecture of Xishuangbanna. It harbors six genera of ten species, belonging to *Indochinamon* (5 species), *Tenuipotamon* (1 species), *Somanniathelphusa* (2 species), and *Potamiscus* (2 species)*, Mekhongthelphusa* (1 species), and *Tortomon* (1 species)*.* The region is most unique to *Tenuipotamon*, as its distribution is dominated by burrowing on the banks of small stream in the high mountains and has smallest body size than other freshwater crabs (carapace width of the smallest mature individual = 8 mm).

Hotspot 4 (SAMY), situated in the southern Ailao Mountains and lower reaches of Yuanjiang River, includes four genera of nine stream/lake-inhabiting species: *Indochinamon* (6 species), *Parapotamon* (1 species), *Tortomon* (1 species), and *Somanniathelphusa* (1 species). Hotspot 5 (MMWP), located in County-Maguan and County-Malipo County in Wenshan Prefecture, comprises five genera of six species including *Indochinamon* (2 species), *Barbamon* (1 species)*, Lacunipotamon* (1 species)*, Chinapotamon* (1 species), and *Somanniathelphusa* (1 species).

Hotspot 6 (WYS) is located in the Wuyishan Mountains, where ten species in five genera are distributed, including *Bottpotamon* (1 species), *Huananpotamon* (1 species), *Minpotamon* (2 species), *Nanhaipotamon* (3 species), and *Sinopotamon* (3 species). The first three genera are smaller in carapace-size and are found under debris in streams or in burrows along banks. The carapace of *Sinopotamon* and *Nanhaipotamon* are relatively larger but occupy very different habitats: *Sinopotamon* seeks shelter under rocks in streams, while *Nanhaipotamon* burrows in mud along the banks.

Hotspot 7 (KAGG) is characterized by unique karst areas in Guangxi and Guizhou, where 13 species in six genera are distributed, including *Chinapotamon* (6 species), *Heterochelamon* (3 species), *Indochinamon* (1 species), *Qianguimon* (2 species), *Sinolapotamon* (2 species), and *Somanniathelphusa* (2 species). Several species of *Chinapotamon* have been found in the underground rivers of karst caves, while others inhabit mountain streams.

Hotspot 8 (PHM) located in Pearl River Delta, Hong Kong, and Macau, containing *Cantopotamon* (2 species), *Sinolapotamon* (1 species), *Eurusamon* (1 species), *Nanhaipotamon* (4 species), *Megapleonum* (1 species), and *Somanniathelphusa* (2 species).

Hotspot 9 (HI) on southwestern part of Hainan Island encompasses five genera and 11 species, including burrowing (*Hainanpotamon*, 4 species), inhabiting in lowland stream (*Apotamonautes*, 1 species and 4 subspecies, and *Somanniathelphusa*, 3 species), and hiding in the water-filled crevices of tree (*Neotiwaripotamon*, 2 species) and limestone (*Calcipotamon*, 1 species).

Hotspots 10 and 11 on Taiwan Island are predominantly distributed in the northwestern and southern terminus of the Central Mountains in Taiwan (NCMT and SCMT, respectively). Hotspot 10 includes two genera with seven species, namely *Geothelphusa* (6 species) and *Candidiopotamon* (1 species). Meanwhile, Hotspot 11 comprises four genera of 18 species, including *Geothelphusa* (15 species), *Candidiopotamon* (1 species), *Nanhaipotamon* (1 species) and *Somanniathelphusa* (1 species). *Geothelphusa* species inhabit montane streams with relatively large elevation differences in habitat between species. *Somanniathelphusa* is predominantly found in canals next to lowland rice paddies, while *Nanhaipotamon* typically burrows along the stream banks. The *Candidiopotamon* species are primarily distributed on both sides of the Central Mountain Range, with a wide range of distribution.
